# Genome-Wide Association Study for Markers Related to Protein, Fiber (ADF and NDF) and Oil Content in Winter Oilseed Rape Seeds (*Brassica napus* L.)

**DOI:** 10.3390/ijms262411931

**Published:** 2025-12-11

**Authors:** Agnieszka Łopatyńska, Joanna Wolko, Łukasz Wolko, Jan Bocianowski, Julia Spychała, Aleksandra Noweiska

**Affiliations:** 1 Plant Breeding and Acclimatization Institute—National Research Institute in Radzików, 05-870 Błonie, Poland; j.wolko@ihar.edu.pl (J.W.); j.spychala@ihar.edu.pl (J.S.); a.noweiska@ihar.edu.pl (A.N.); 2 Department of Biochemistry and Biotechnology, Poznań University of Life Sciences, Dojazd 11, 60-632 Poznań, Poland; lukasz.wolko@up.poznan.pl; 3 Department of Mathematical and Statistical Methods, Poznań University of Life Sciences, Wojska Polskiego 28, 60-637 Poznań, Poland; jan.bocianowski@up.poznan.pl

**Keywords:** association mapping, rapeseed quality, seed biochemical components, SNP markers

## Abstract

Seed biochemical composition critically influences the quality and industrial value of oilseed rape (*Brassica napus* L.). Understanding the genetic basis of seed oil, protein, and fiber content is essential for breeding improved cultivars. Here we conducted a genome-wide association study (GWAS) on 350 diverse winter oilseed rape lines over three years, using near-infrared reflectance spectroscopy (NIRS) to measure seed traits and SNP genotyping for association mapping. We identified numerous SNP markers significantly associated with seed oil, protein, acid detergent fiber (ADF), and neutral detergent fiber (NDF) content. From 18,566 detected SNPs, 3782 met stringent criteria and were used for association mapping, resulting in 3189 significant associations across three years. The highest number of associations was observed for protein (3480), followed by NDF (3662), ADF (3422), and oil (2046). Individual markers explained up to 35% of phenotypic variation, indicating strong genetic control of these traits. Gene ontology enrichment analyses linked candidate genes to key metabolic and regulatory pathways influencing these traits: protein biosynthesis and post-translational modification, lipid metabolism regulated by phosphorylation, and transcriptional control of cell wall polysaccharide synthesis. These findings provide valuable molecular markers that can be validated for further use in marker-assisted selection, supporting the development of rapeseed cultivars with optimized seed quality for food, feed, and industrial applications.

## 1. Introduction

Oilseed rape (*Brassica napus* L.; AACC, 2 n = 38) is an essential crop for vegetable oil production worldwide, with Canada, China, India, Australia, France, Germany and Poland as major producers for seeds and oil [1,2]. The value of rapeseed depends mainly on the high oil content in its seeds, which makes rapeseed a significant raw material for the oil and feed industries [3,4]. According to USDA forecasts, rapeseed will remain a key component of sustainable agri-food systems between 2025 and 2035. Rising demand for vegetable oils and feed protein strengthens its role in global food security. Moreover, rapeseed oil is expected to continue as a major raw material for first-generation biofuels, which will dominate the market during this period [4].

The composition of any crop including oilseed rape depends on agroclimatic conditions. Different cultivars exhibit significant variation in oil content and composition. Genetic modifications and plant breeding techniques have produced several oilseed rape cultivars containing different amounts of glucosinolates and other anti-nutritional factors [5]. In general, oilseed rape seeds contain approximately 40–45% oil, 19–28% crude protein, 14–15% crude fiber, 5–6% ash content, 8–10% soluble sugars and 3–4.5% water [5,6,7]. The 00-quality rapeseed oil contains large amounts of C18 fatty acids, including monounsaturated oleic acid (C18:1, ~60% by volume), as well as moderate amounts of polyunsaturated linoleic acid (C18:2, ~30% by volume) and linolenic acid (C18:3, ~10% by volume). Besides high quality oil, oilseed rape meal after oil extraction also provides protein-rich feed for animals [8].

Seeds from species such as soybeans, oilseed rape, cotton, sunflower and peanuts are the most abundant for protein meal, accounting for 69%, 12.4%, 6.9%, 5.3% and 2.8% of global protein meal production, respectively [9]. Plant proteins from oilseeds and legumes has become an alternative to animal proteins [10]. Moreover, recent studies have explored the potential of using rapeseed protein concentrate as a new protein source for meat analogs [11,12]. Proteins are crucial to human and animal nutrition—they consist of amino acids and are essential for key metabolic processes [13]. Notably, rapeseed protein is rich in sulfur-containing amino acids—cysteine and methionine (3.0–4.0% of total protein)—meeting FAO requirements for human nutrition more closely than other high-quality plant proteins [14,15]. De-oiled meal contains up to 50% protein (on a dry matter basis). Oilseed rape protein is mainly composed of storage proteins, namely cruciferin and napin, which account for about 60% and 20% of all proteins, respectively [16,17].

Fiber content in seeds is the main factor responsible for reducing the digestibility and energy value of oilseed rape meal [18]. Neutral detergent fiber (NDF) and acid detergent fiber (ADF) are key measures used in feed analysis. NDF includes hemicellulose, cellulose, and lignin, while ADF is a subset of NDF containing only cellulose and lignin and is a better indicator of feed digestibility and energy content. In oilseed rape, the fiber components are strongly correlated with seed phenolic compounds and seed color [19,20,21]. Studies of yellow-seeded canola have been conducted to reduce the dietary fiber content of the seeds while increasing the oil and protein content [21]. Phenolic compounds, integral to dietary fiber and products of phenylpropanoid and flavonoid pathways, also influence oil quality, meal value, and seed color [20,22,23]. Low fiber content, particularly acid detergent lignin (ADL) content, was significantly correlated with seed color [20,22] due to lignin and pigment synthesis sharing the same precursors.

Combinations of different traits to develop cultivars tailored for specific applications of this crop remains a key priority in oilseed rape research and breeding programs around the world [24]. Moreover, integration of the results obtained by genotyping and phenotyping of plants is crucial, because it allows to select potential candidate genes determining traits valuable in oilseed rape processing. To meet the growing demand in oilseed breeding programs, increasing seed oil content and oil production per unit area is essential. Although a comprehensive review of the biological and metabolic pathways of triacylglycerol synthesis has been well recorded, the genetic and molecular mechanisms underlying oil accumulation in *B. napus* seeds remain poorly understood [25,26].

Nowadays, genetic studies focused on the biochemical content of seeds are increasingly carried out using modern molecular biology tools that allow the identification of genes and molecular markers associated with desirable seed quality traits. One of the methods used in genetic research on oilseed rape seed quality is genome-wide association studies (GWAS) [27], which allow the identification of loci associated with quantitative traits such as fat, protein or glucosinolate content. Thanks to large genetically diverse populations and the use of dense SNP maps, it is possible to detect markers closely linked to genes responsible for the metabolism of lipids, proteins or anti-nutritional compounds [28,29]. Furthermore, the integration of genotype and phenotype data within functional genomics enables a better understanding of the mechanisms regulating the biosynthesis of fats, proteins and anti-nutritional compounds in rapeseed. This knowledge supports the development of precise breeding strategies, including genomic selection, which uses whole-genome SNP profiles to predict plant breeding values [30].

The aim of the study was to identify SNP markers associated with seed oil, protein and fiber content through association mapping of a collection of 350 differentiated winter oilseed rape lines in a three-year field experiment. Therefore, we performed association and physical mapping of the identified SNP markers to verify the function and role of the genes associated with them.

## 2. Results

### 2.1. Phenotyping

**Figure 1:**
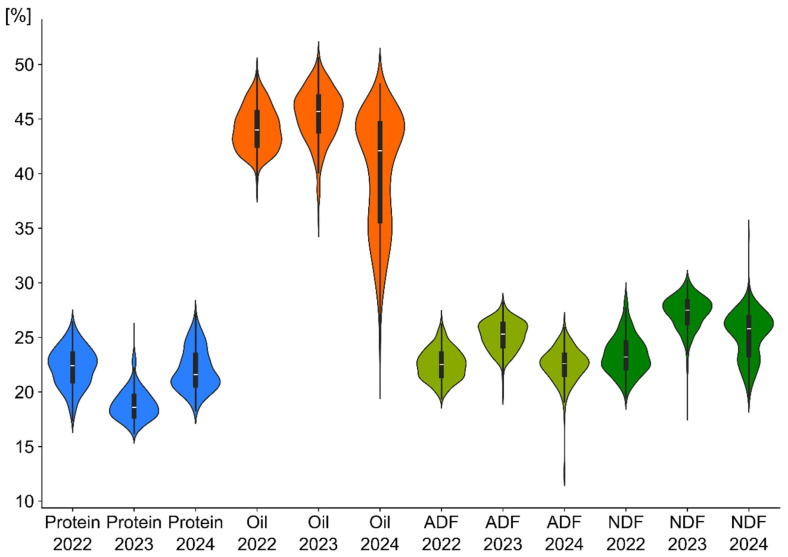
Density charts reveal the distributions of protein, oil, ADF (acid detergent fiber), and NDF (neutral detergent fiber) traits in the specified years.

The empirical distributions of all four quantitative traits conformed to the normal distribution. Bartlett’s test confirmed the homogeneity of variances across all four traits. The phenotypic variations for oil, protein, ADF and NDF content of 350 rapeseed accessions were measured and are presented in Figure 1. Analysis of variance indicated that the main effects of year and line, as well as year × line interaction were significant for all the traits of the study. The average protein content in 2023 was the lowest, resulting in higher oil content and ADF and NDF. In 2024, the average oil content in the tested genotypes was significantly lower compared to previous years (2022, 2023), but no such trend was observed in the other tested traits.

### 2.2. Genotyping

**Figure 2:**
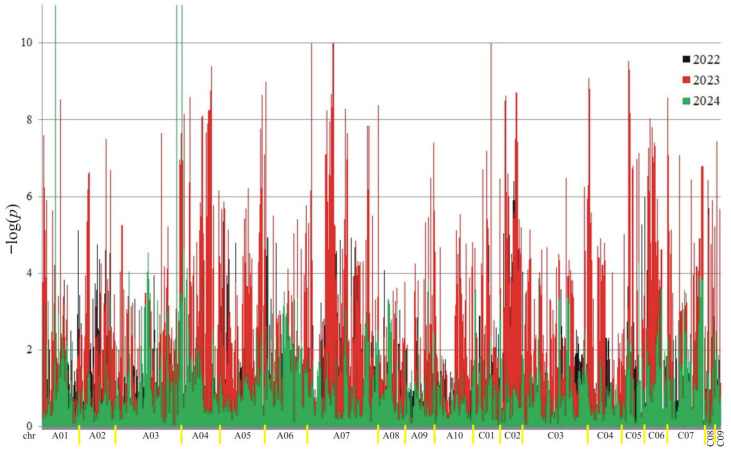
Manhattan plot for association analysis of protein in collection of 350 rapeseed accessions. The colors of the peaks on the graph indicate the individual years of the experiment: black (2022), red (2023), and green (2024).

**Figure 3:**
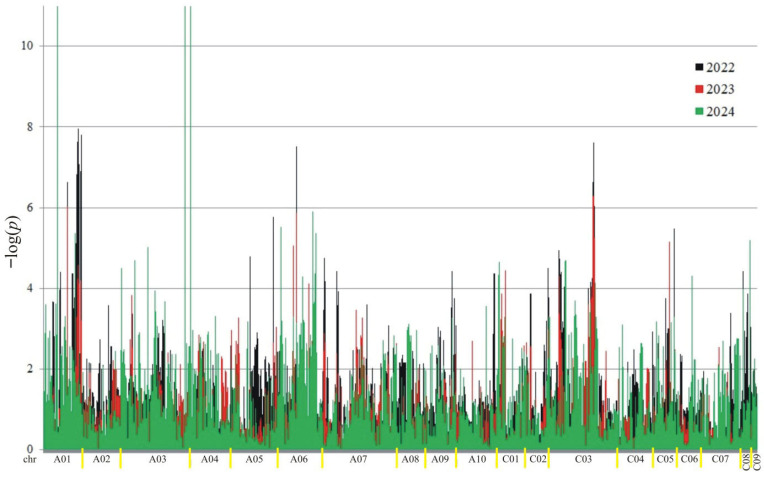
Manhattan plot for association analysis of oil in collection of 350 rapeseed accessions. The colors of the peaks on the graph indicate the individual years of the experiment: black (2022), red (2023), and green (2024).

**Figure 4:**
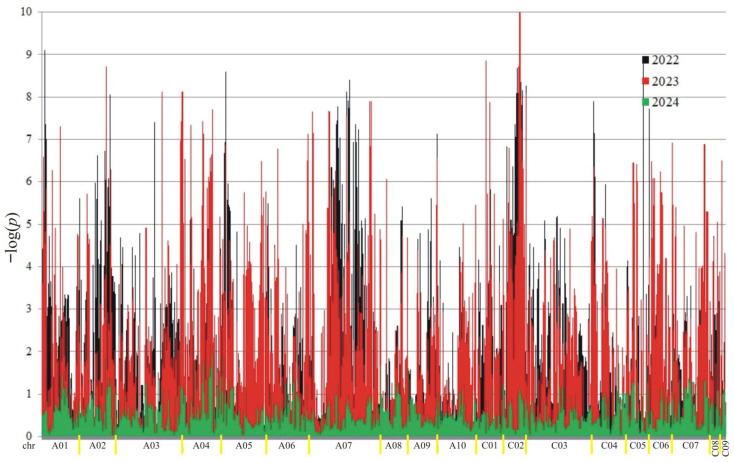
Manhattan plot for association analysis of ADF in collection of 350 rapeseed accessions. The colors of the peaks on the graph indicate the individual years of the experiment: black (2022), red (2023), and green (2024).

**Figure 5:**
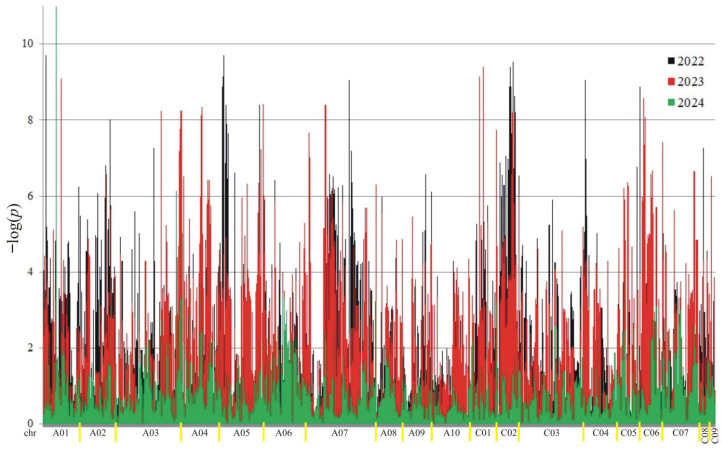
Manhattan plot for association analysis of NDF in collection of 350 rapeseed accessions. The colors of the peaks on the graph indicate the individual years of the experiment: black (2022), red (2023), and green (2024).

A total of 18,566 putative SNPs were detected. For association mapping, 3782 meeting the criteria (MAF > 0.25 and the number of missing observations < 10%) were used. Of these SNPs, 3189 were associated (at the 0.05 level) with at least one trait in at least one year (Figure 2, Figure 3, Figure 4 and Figure 5).

The number of significant markers associated with each trait is presented in Table 1. The highest number of associations was observed for protein (3480), and the lowest for oil (2046). The distribution of associations differed across the years. In the first year (2022), the most associations were observed for oil and ADF, while in the second year, they were highest for protein and NDF. In the last year of the study (2024), the fewest associations were observed for protein, ADF, and NDF.

The percentages of variation in observed traits explained by individual markers ranged from 0.8% to 35.0% (Table 2). The most important SNPs with the highest explained variance are included in the Supplementary Tables S1 and S2.

### 2.3. Genome-Wide Association Study Identified Key Markers Associated with Seed Composition Traits

**Figure 6:**
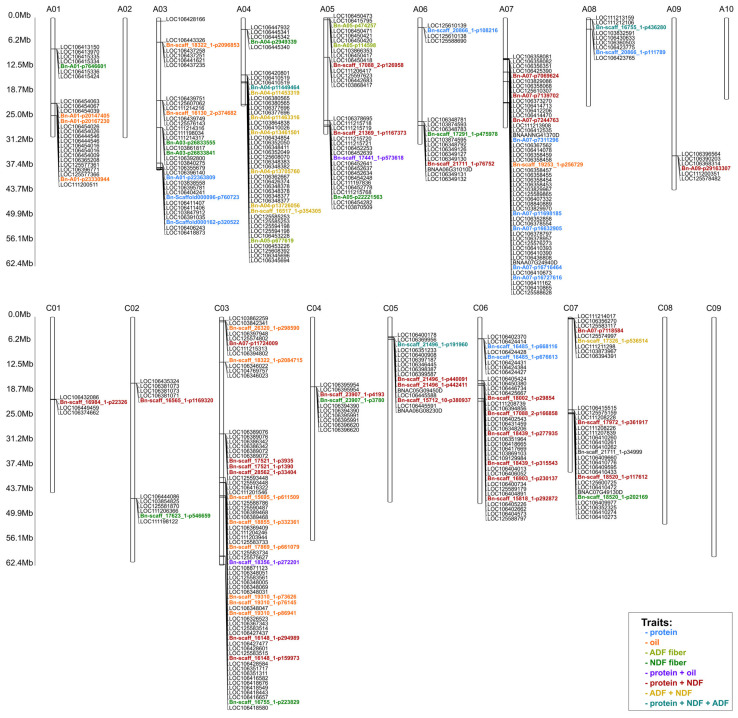
The location of SNP with mapped candidate genes and the abbreviation “Mb” next to the scale indicates the Megabase unit referring to the physical length of the chromosomes. The legend shows colors representing markers associated with the studied traits or combinations of studied traits.

The detected associations were linked to one, two, three, and four traits simultaneously. Eighty-one SNP markers with the highest LOD values and significant in all years of the study were selected for further analysis (Supplementary Tables S1 and S2). A total of 12 SNPs markers were chosen for protein, 14 for oil, three for ADF, and 11 for NDF (Supplementary Table S1). For protein and NDF traits, 29 SNPs markers were selected; for ADF and NDF seven SNPs markers were selected; for protein and oil content trait—two SNPs markers; and for three traits: protein, ADF and NDF, three SNPs markers were selected (Supplementary Table S2). Selected markers were mapped using BnIR databease (https://yanglab.hzau.edu.cn/BnIR/jbrowse (accessed on 3 March 2025)) using *B. napus* Express617 v1 as the reference genome and genes located near the SNP markers were selected (Supplementary Table S3). Figure 6 shows the locations of selected candidate genes.

Simultaneously, for the ADF + NDF traits, the markers/genes are mainly located on chromosome A04. For the ADF trait, the markers/genes are located in the distal part of the short arm of A05, while for NDF they are distributed on chromosomes A and C of the genome. For the oil trait, markers/genes are mainly located in genome A and chromosome C03. Similarly, for the protein trait, genes are located in genome A and chromosome C06. The associated markers/genes for the protein + oil trait are located on chromosomes A05 and C03. Interestingly, for protein + NDF, most SNPs are located in genome C. However, for the three traits (protein + ADF + NDF), associations with genes/markers located on chromosomes A04 and C05 have been demonstrated.

### 2.4. Gene Enrichment Analysis

**Figure 7:**
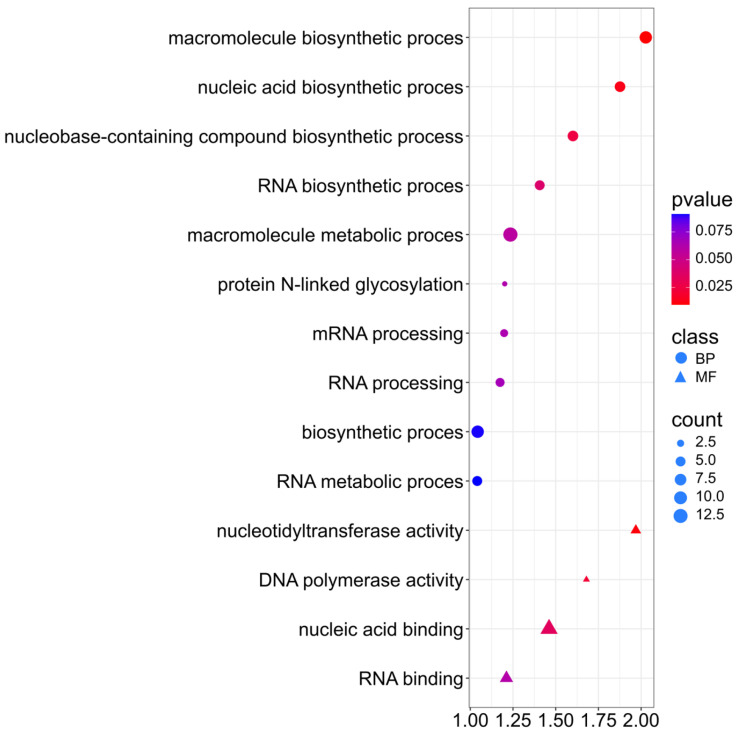
Gene Ontology (GO) pathway enrichment analysis for protein. GO enrichment functions that were significantly enriched in biological processes (BP) and molecular functions (MF) were sorted by *p*-value.

A Go Ontology (GO) analysis was performed on selected genes using the *p*-value test (Supplementary Materials S4). For the protein trait, genes were associated with the Biological Process and Molecular Function classes (Supplementary Materials S4). For Biological Process, the genes mainly indicate involvement in macromolecule biosynthetic process (GO:0009059), nucleic acid biosynthetic process (GO:0141187), and nucleobase-containing compound biosynthetic process (GO:0034654). GO terms may indicate that the protein content in rapeseed seeds is associated with genes related to the activity of nucleic acid biosynthesis and translation processes. For Molecular Function, three genes are involved in nucleotidyl transferase activity (GO:0016779) (Figure 7). In addition, (GO:0032774) RNA biosynthetic process, (GO:0006397) mRNA processing, and (GO:0006396) RNA processing indicate involvement in post-translational processing and regulation of protein-coding genes. (GO:0006487) Protein N-linked glycosylation may indicate the activity of post-transcriptional processes affecting the stability and functionality of storage proteins in seeds.

**Figure 8:**
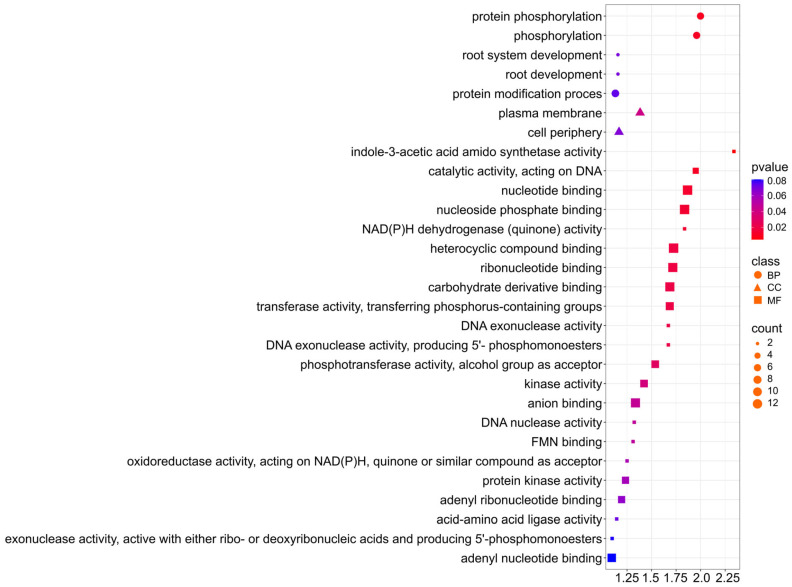
Gene Ontology (GO) pathway enrichment analysis for oil. GO enrichment functions that were significantly enriched in biological processes (BP), cellular components (CC), and molecular functions (MF) were sorted by *p*-value.

For the oil trait, the selected genes are involved in Biological Process, Cellular Component, and Molecular Function (Figure 8; Supplementary Materials S4). With a *p*-value < 0.02, six genes are involved in protein phosphorylation (GO:0006468) and phosphorylation (GO:0016310), indicating that they are one of the major regulatory mechanisms for lipid metabolism. The terms (GO:0016301) kinase activity, (GO:0016772) transferase activity, transferring phosphorus-containing groups, (GO:0004672) and protein kinase activity also provide information for the role of phosphorylation in the control of fatty acid and triacylglycerol (TAG) biosynthesis. Furthermore, the identification of (GO:0010279) indole-3-acetic acid amido synthetase activity suggests a correlation with hormonal regulation, particularly auxin metabolism and lipid synthesis. For Cellular Component, six genes are associated with the plasma membrane (GO:0005886) and cell periphery (GO:0071944), suggesting the contribution of membrane proteins in the transport of lipids and fatty acid precursors.

**Figure 9:**
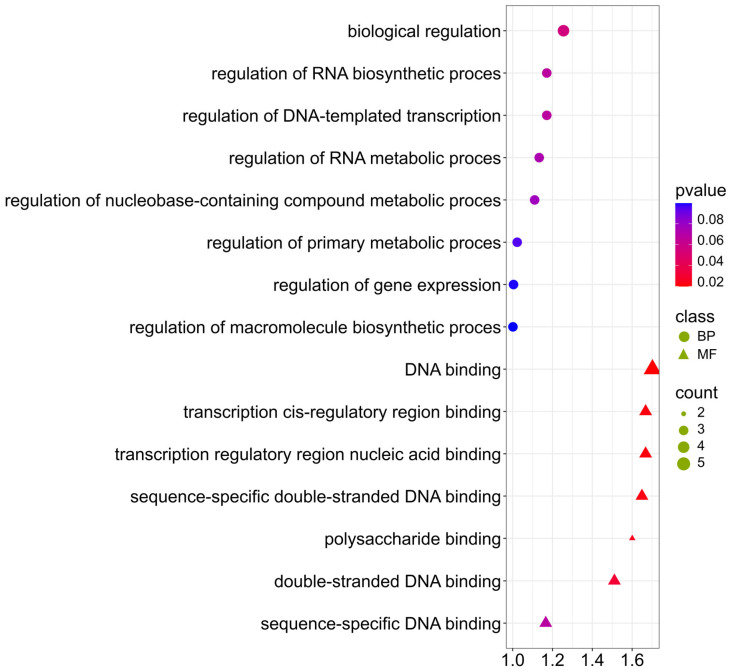
Gene Ontology (GO) pathway enrichment analysis for ADF. GO enrichment functions that were significantly enriched in biological processes (BP) and molecular functions (MF) were sorted by *p*-value.

The genes selected for the ADF trait are mainly involved in biological processes and molecular functions (Figure 9; Supplementary Materials S4). Terms obtained: (GO:0006355) DNA-based transcription regulation, (GO:0065007) biological regulation, (GO:0010468) gene expression regulation, (GO:0003677) DNA binding, (GO:0000976) binding of the cis-regulatory region of transcription, (GO:1990837) sequence-specific double-stranded DNA binding, may indicate that the content of ADF can be regulated by transcriptional mechanisms and suggests the involvement of transcription factors in the regulation of the biosynthesis of polysaccharides, such as cellulose and lignin, the main components of ADF. Transcription factors may regulate the activity of genes encoding enzymes involved in lignification and cell wall biosynthesis. It is also worth mentioning term (GO:0030247) polysaccharide binding, which indicates the presence of proteins interacting with polysaccharides potentially involved in the formation or modification of the cell wall structure.

**Figure 10:**
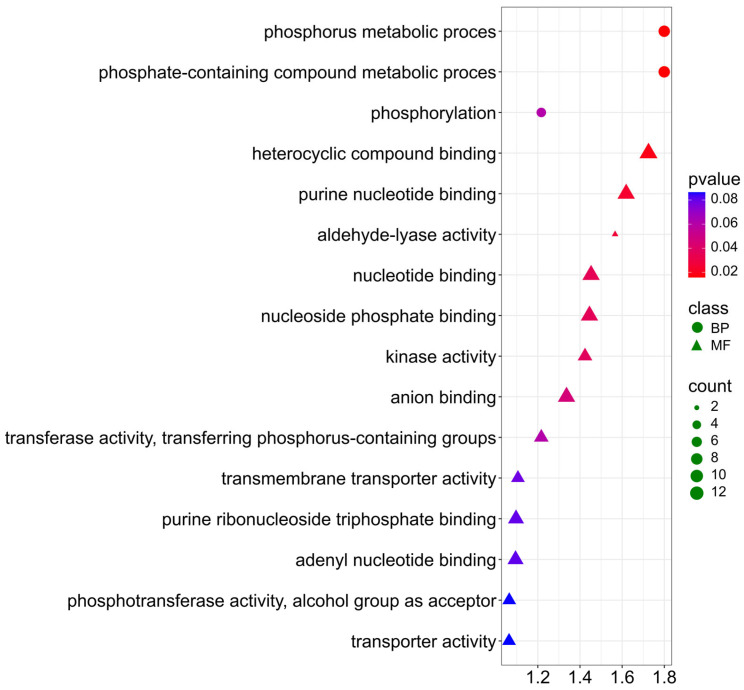
Gene Ontology (GO) pathway enrichment analysis for NDF. GO enrichment functions that were significantly enriched in biological processes (BP) and molecular functions (MF) were sorted by *p*-value.

For the NDF trait, genes are similarly involved in biological processes and molecular functions (Figure 10; Supplementary Materials S4). In BP, they are mainly involved in the following processes: phosphorus metabolic process (GO:0006793), phosphate-containing compound metabolic process (GO:0006796) involved in the regulation of biosynthesis and modification of cell wall polysaccharides—the main components of the NDF fraction. Additionally, terms: (GO:0016310) phosphorylation, (GO:0016301) kinase activity, (GO:0016772) transferase activity, transferring phosphorus-containing groups, indicating involvement in phosphorylation and regulation of structural carbohydrate metabolism. For molecular function, genes participate with a *p*-value < 0.03 in heterocyclic compound binding (GO:1901363), purine nucleotide binding (GO:0017076), and aldehyde-lyase activity (GO:0016832).

**Figure 11:**
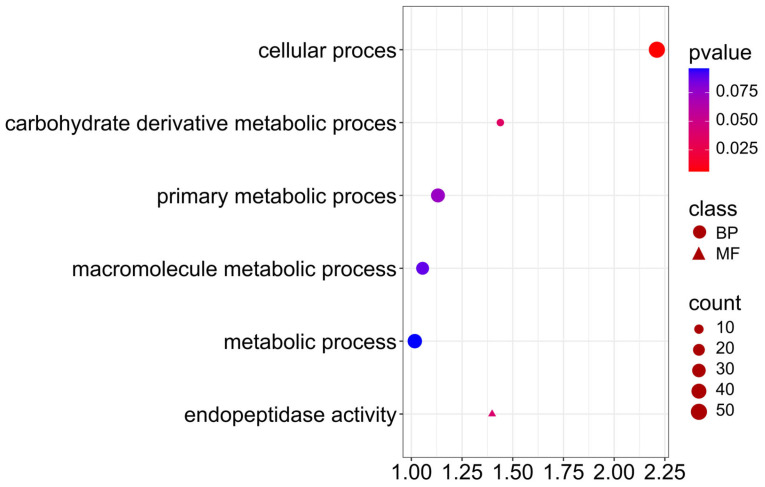
Gene Ontology (GO) pathway enrichment analysis for protein and NDF. GO enrichment functions that were significantly enriched in biological processes (BP) and molecular functions (MF) were sorted by *p*-value.

Candidate genes associated with protein and NDF content trait (Figure 11; Supplementary Materials S4), participate in cellular process (GO:0009987), carbohydrate derivative metabolic process (GO:1901135), indicating the activity of cellular processes related to the metabolism of carbohydrate derivatives, which are precursors of the structural proteins and cell wall components. Interestingly, five genes are also involved in endopeptidase activity (GO:0004175), indicating the involvement of proteolytic enzymes in protein remodeling during seed maturation. Endopeptidase activity may be important in nitrogen accumulation and reuse in cell wall synthesis, which may influence differences in protein and NDF fraction contents.

**Figure 12:**
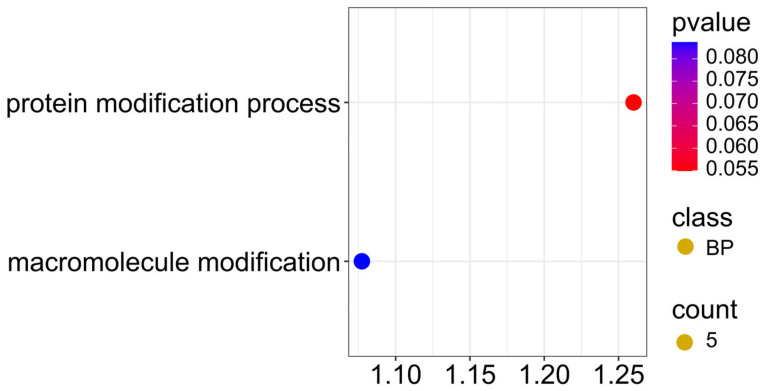
Gene ontology (GO) pathway enrichment analysis for ADF and NDF. GO enrichment functions that were significantly enriched in biological processes (BP) were sorted by *p*-value.

Five candidate genes located in the region of SNPs associated with two traits simultaneously, ADF and NDF (Figure 12; Supplementary Materials S4), showed involvement in protein modification (GO:0036211) and macromolecule modification (GO:0043412) processes in the GO enrichment analysis, with a *p*-value < 0.08, indicating that post-translational modification processes of proteins play a key role in regulating the structural composition of seeds.

**Figure 13:**
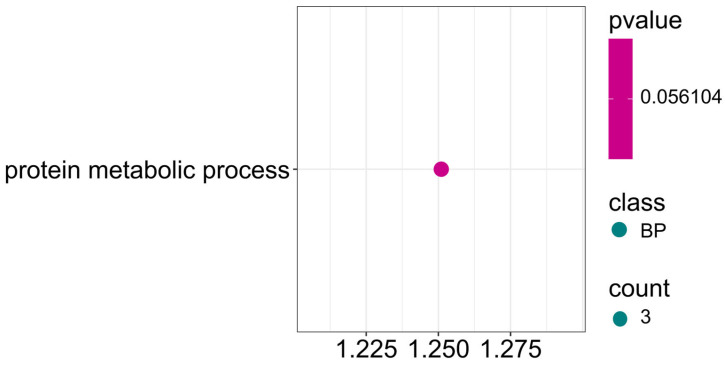
Gene ontology (GO) pathway enrichment analysis for protein, ADF and NDF. GO enrichment function that was significantly enriched in biological processes (BP) was presented by *p*-value.

Among the identified markers, some SNPs also showed associations for three traits simultaneously: protein, ADF, and NDF (Figure 13; Supplementary Materials S4). Three candidate genes are involved in the protein metabolic process (GO:0019538) with a *p*-value approximately 0.06. These results suggest that differential seed composition in terms of protein content and ADF and NDF fiber fractions is closely related to the activity of metabolic pathways controlling protein synthesis, modification, and protein degradation.

## 3. Discussion

According to the OECD-FAO agricultural outlook for 2025–2034, demand for vegetable oils in the food industry will continue to grow strongly, partly due to population growth. Current forecasts indicate that soybean, oil palm and other major oil crops, including sunflower, groundnut, rapeseed, and mustard, are likely to continue to expand in global food supplies [31,32]. Oilseed rape belongs to the group of industrial crops grown mainly for oil extraction. Seeds of rapeseed with appropriate technological quality can be processed for food or technical purposes. The processing of rapeseed is mainly aimed at oil extraction, while the resulting by-products include pomace (rapeseed cake) and post-extraction meal. Subsequently, these resources are rich in protein and are suitable for livestock feed purposes [33]. Therefore, genetic research focused on genes determining functional traits of oilseed rape seeds consistently seems to be crucial and required.

The key objectives that will form the basis for crop development in different environments are understanding and applying genetic control of important agronomic traits in rapeseed, such as seed oil composition and concentration, as well as interactions between these traits and the environment [34]. Over the past two decades, rapeseed with a high oleic acid content (over 75% C18:1 in seed oil) and low linolenic acid content (less than 3% C18:3) (HOLL) has been widely used. Such oil is optimal for long-term storage and exhibits high thermostability thanks to its high resistance to oxidation and reduced formation of harmful trans isomers during raw material processing [35]. In recent decades, many research results have been reported concerning the genes and metabolic pathways that determine the quantity and quality of oil in rapeseed. In plants of the *Brassica* genus, including winter rapeseed, *FAD* genes are among the best-known determinants of fatty acid composition. The *FAD* (Fatty Acid Desaturase) family includes a group of specialized enzymes that catalyze desaturation reactions, i.e., the introduction of double bonds into fatty acid chains. This process is one of the most important stages of lipid modification, as it determines membrane fluidity, the TAG (triacylglycerol) fatty acid profile, and ultimately the utility and quality of rapeseed oil. The major locus for high C18:1 was proven to be the fatty acid desaturase-2 (*FAD2*) gene. It has been shown that high oleic acid (C18:1) and low linolenic acid (C18:3) content can be mainly linked to the presence of the *FAD2* (for C18:1) and *FAD3* (for C18:3) alleles [36]. In our study, for the trait of oil content in seeds, the identified markers/genes are mainly located in genome A and chromosome C03 (Figure 6). Based on LOD values, the following statistically significant markers were identified for this trait: three SNP markers in chromosome A01, two SNP markers in chromosome A03, one SNP marker in chromosome A07, and as many as eight SNPs in chromosome C03 (Figure 6). Moreover, our in-depth GO analysis revealed that the selected genes involved in shaping the oil content trait in seeds are involved in biological processes, cellular components, and molecular functions. At a *p*-value < 0.02, six genes are involved in protein phosphorylation (GO:0006468) and phosphorylation (GO:0016310), indicating that they are one of the main mechanisms regulating lipid metabolism. The terms (GO:0016301) kinase activity, (GO:0016772) transferase activity, transfer of phosphorus-containing groups, (GO:0004672) and protein kinase activity also provide information on the role of phosphorylation in the control of fatty acid and triacylglycerol (TAG) biosynthesis. Furthermore, the identification of indole-3-acetic acid amide synthase activity (GO:0010279) suggests a correlation with hormonal regulation, in particular auxin metabolism and lipid synthesis. In terms of cellular components, six genes are located in the cell membrane (GO:0005886) and at the cell periphery (GO:0071944), suggesting the involvement of membrane proteins in the transport of lipids and fatty acid precursors (Figure 8).

Protein content is often negatively correlated with oil content in seeds, which is a classic breeding problem. Many QTL and GWAS show that some QTL simultaneously influence oil and protein content, but often in an antagonistic manner. For breeding, this indicates that improving one trait commonly leads to a decrease in another. Triacylglycerols and storage proteins (e.g., napin and cruciferin) are the main components of energy reserves in seeds. Both are synthesized from the same carbon and energy precursors, which is mainly sucrose.

The biosynthesis of oil and protein during embryo development is a complex and hierarchical biological process regulated by certain transcription factors and complex networks of gene interactions [37,38]. In the literature data, one of the most recent works in this field is the study by Chao et al. [39]; which was aimed at the QTLs responsible for the variability of oil and protein content in rapeseed, taking into account their interdependence and stability in different environments [39]. They identified 67 QTLs for oil content and 38 QTLs for protein, 11 of which were common to both traits, often showing opposite effects of alleles confirming the genetic antagonism between oil (SOC) and protein (SPC) content in seed. Some of these QTLs are mainly located on chromosomes: A03, A09, C03 and C05. In the present study, for the seed protein content trait, the markers are mainly located in genome A. Based on the LOD values, the following markers were identified for this trait: three SNP markers in the distal part of chromosome A03, one SNP in chromosome A06, five SNPs in chromosome A07, one SNP in A08, and two SNP markers in chromosome C06 (Figure 6). In Chao’s study candidate genes in QTL regions include those involved in fatty acid metabolism and lipid biosynthesis (e.g., TAG, FA elongation, acyl-lipid metabolism enzymes). In particular, the regions on chromosome C05, comprising *qOC-C5-11* (for SOC) and *qPC-C5-5* (for SPC), contain a common gene *IPGAM2*, encoding the 2,3-bisphosphoglycerate-independent phosphoglycerate mutase, which participates in the conversion of glycolysis metabolites and affects the availability of precursors for both biosynthetic pathways. In addition, orthologs of key glycolysis enzymes such as *HKL*, *PFK*, *GAPCP*, and *PGK*, which are responsible for the conversion of glucose to pyruvate, a compound that is a central metabolic hub for the synthesis of both fats and amino acids, were identified in the overlapping QTLs. A total of 58 orthologs were common to SOC- and SPC-QTLs, confirming that basic carbohydrate metabolism plays an essential role in the simultaneous provision of precursors for oil and protein synthesis during embryo development [39]. In our study, based on LOD values, the markers Bn-scaff_17441_1-p573618 (chromosome A05) and Bn-scaff_18356_1-p272201 (chromosome C03) were associated with both oil and protein content (Figure 6). However, our GO analyses of genes in the region of these SNP markers did not show a statistically significant association with the metabolic processes and pathways of protein and oil synthesis in seeds. Nevertheless, further in-depth analysis of these regions on both chromosomes is indicated.

The reduction in seed oil content at late maturity is due to two aspects. The first is that the gene expression involved in oil synthesis is significantly decreased. In the second way, a large number of genes involved in oil degradation are being expressed and are active during this stage [40]. In a GWAS analysis by Wang et al. [41], they identified the *BnPTL* gene (BnaC07g30920D), a patatin-like lipase (*PTL*) gene that was associated with seed oil content in oilseed rape. The scientists showed that six SNPs in the promoter region of the BnaC07g30920D gene were associated with a significant reduction in seed oil, leading to a reduction in oil content by as much as 4.7–6.2% [41]. In the study of Xiao et al. (2019) [26], researchers used GWAS analysis on 588 rapeseed lines, identifying 17 SNP loci significantly associated with oil content. Additionally, transcriptomic analysis revealed seven candidate genes associated with oil accumulation [26].

Another approach to studying the genetic basis of oil content in seeds is based on the assumption that lipid accumulation is not uniform in all tissues of rapeseed [42]. Instead of studying whole rapeseeds, the authors divided the seeds into four tissues: seed coat (SC), outer cotyledon (OC), inner cotyledon (IC), and radicle (R). The MRI (magnetic resonance imaging) technology was used for non-invasive 3D imaging and quantitative analysis of lipids in individual tissues. Subsequently, a dense genetic map was constructed with a population of 200 doubled haploid lines (DH). QTL mapping for oil traits in different tissues was performed, and transcriptomic analysis was carried out at different stages of seed development (24 and 33 days after flowering) in two parent varieties differing in oil content. As a result, many QTLs were identified, including new tissue-specific ones, as well as candidate genes whose expression and location correspond to QTL [42]. Many of the QTLs were tissue-specific (i.e., affecting only one tissue). Fourteen such unique loci were identified, seven of which are completely new compared to previous studies. The authors [42] identified 86 candidate genes associated with lipids, which are located inQTL intervals for various tissues studied, including the *CAC2* gene (an enzyme that limits the rate of fatty acid synthesis) in QTLs for OC and IC tissues; genes for FA elongation (KCS17, FAE1), TAG synthases (e.g., LPAT, DGAT).

The protein in the rapeseed pomace remaining after pressing canola oil could be widely used if the seeds had a lower fiber content, since fiber reduces the energy value of the pressed oil. A solution to this issue could be to breed cultivars with yellow seeds due to their thinner seed coat and lower concentration of polyphenolic compounds, while having higher protein and fat content. It is worth noting that these traits are highly desirable and reflect the effectiveness of breeding aimed at improving oilseed rape quality traits [43]. In the study by Shi et al. [44], the authors analyzed crude fiber components (including NDF) in *B. napus* shoots and performed a GWAS analysis on a panel of 202 rapeseed cultivars to analyze the traits: NDF, ADF, ADL, hemicellulose, and cellulose. The study identified 1285 significant SNPs and 97 regions associated with fiber components. In addition, seven candidate genes were identified on chromosomes A02, A08, A09, and C09 associated with CF (crude fiber) traits [44]. In a study conducted by Gacek et al. [45] the genetic and molecular mechanisms regulating the synthesis of seed storage proteins (SSPs) in *B. napus* seeds was analyzed, including the main fractions: cruciferin and napin—responsible for the protein content and quality of rapeseed. The authors showed that the accumulation of SSPs is tightly controlled by a network of transcription factors, such as LEC1, LEC2, FUS3, and ABI3, and occurs mainly during the middle phase of seed development. In their study, the researchers emphasized the existence of a negative correlation between protein and oil content in rapeseed, which results from metabolic competition for common substrates and energy during seed maturation [45]. The same relationship between these traits was visible in our study—if the identified SNP marker was positively associated with protein content, it was negatively associated with oil content and vice versa.

At the cellular level in plants, protein phosphorylation and lipid metabolism in plants are interrelated because protein phosphorylation (the addition of a phosphate group to a protein) regulates many processes, including lipid metabolic pathways. It regulates enzymes involved in lipid synthesis and degradation, influences the storage and transport of energy (ATP) necessary for these processes, and may respond to external signals regarding nutrient availability. Understanding this relationship is key to optimizing plant growth and development. Protein phosphorylation activates or deactivates enzymes crucial for lipid metabolism, such as those involved in the synthesis of fatty acids and triacylglycerols [46,47]. In this study, we have shown that six candidate genes are involved in protein phosphorylation (GO:0006468) and phosphorylation (GO:0016310), indicating that they are one of the major regulatory mechanisms for lipid metabolism. The terms: (GO:0016301) kinase activity, (GO:0016772) transferase activity, transferring phosphorus-containing groups, (GO:0004672) and protein kinase activity, also provide information for the role of phosphorylation in the control of fatty acid and triacyl-glycerol (TAG) biosynthesis (Supplementary Materials S4).

A study by Suprianto [48] analyzed the genetic variability of fiber fraction content (NDF, ADF, ADL) in winter rapeseed seeds and their dependence on protein and oil content. The aim of the study was to determine the heritability and breeding potential of these traits in rapeseed populations. High heritability of fiber content and significant differences between genotypes were demonstrated. A negative correlation was found between fiber and oil content, while the relationship between fiber and protein was weaker and variable. The author emphasized that the selection of genotypes with reduced fiber content could improve the quality and feed value of rapeseed meal [48]. In our study, candidate genes associated with protein and NDF traits simultaneously, presented in Figure 11, participate in cellular process (GO:0009987), carbohydrate derivative metabolic process (GO:1901135), indicating the activity of cellular processes related to the metabolism of carbohydrate derivatives, which are precursors of the structural proteins and cell wall components. Interestingly, five genes are also involved in endopeptidase activity (GO:0004175) (Supplementary Materials S4). The GO-enrichment analysis also selected candidate genes associated with both traits: protein and NDF fiber, presented in Figure 11, which participate in cellular process (GO:0009987) and carbohydrate derivative metabolic process (GO:1901135), indicating the activity of cellular processes related to the metabolism of carbohydrate derivatives, which are precursors of the structural proteins and cell wall components. Interestingly, five genes are also involved in endopeptidase activity (GO:0004175), indicating the involvement of proteolytic enzymes in protein remodeling during seed maturation. Endopeptidase activity may be important in nitrogen accumulation and reuse in cell wall synthesis, which may influence differences in protein and NDF fraction contents. Notably, there is a lack of studies reporting whole-genome analyses of fiber content in rapeseed or related species. This knowledge gap highlights the critical need for further genomic investigations to elucidate the genetic basis of fiber accumulation in seeds.

## 4. Materials and Methods

### 4.1. Winter Oilseed Rape Germplasm Diversity Panel and Field Experience

The collection of plant material represented 350 diverse population lines accessions of winter oilseed rape were cultivated in Strzelce Plant Breeding Ltd. (Strzelce, Poland) IHAR Group in Borowo Division, for three-years field experiment (2022–2024). The tested population lines were selected through breeding experiments conducted by the company Strzelce Plant Breeding, IHAR Group, and examined for their yield value. The names of the rapeseed lines studied are presented in Supplementary Table S1. The experiment was conducted using a system with random patterns and two test replicates each year. The experimental plots had an area of 7.5 m^2^. The density of the plots was 50 plants/m^2^. In crop season, weather conditions were normal for Poland (Table 3). All experiments were conducted in accordance with local field management and cultivation practices, which mainly consisted of insect spraying, fertilization, and weeding in the first stages of growth.

### 4.2. Seed Measurement of Biochemical Compounds by NIRS

Seed composition traits, including oil, protein, and fiber contents, were analyzed to assess genotypic variation. The contents of oil, protein and fiber (ADF and NDF) were determined in the Laboratory of Biochemistry PBAI-NRI in Poznań from each seed sample using near-infrared reflectance spectroscopy (NIRS)—Infratec 1255 Analyser (FOSS, Hillerød, Denmark) [49]. Reference analyses of calibration samples were performed using the following methods: Kjeldahl (for protein content), Soxhlet (for oil content), and Van Soest (for NDF and ADF content). Each analysis was performed in two replicates to reduce the error of the reference samples. Calibration was performed on the basis of approximately 100 samples for NDF and ADF and 50 samples for protein and oil, for the calibration of yellow-seeded and black-seeded rapeseed samples from PBAI-NRI seed collection. The detailed calibration methodology was described by Michalski and Czernik-Kołodziej [49].

### 4.3. Phenotypic Data Analysis

The conformity of the empirical distributions of four analyzed quantitative traits with the normal distribution was assessed using the Shapiro–Wilk *W*-test [50]. Homogeneity of variances was evaluated using Bartlett’s test. Two-way analyses of variance (ANOVA) were conducted to assess the effect of year and line, as well as year-by-line interaction on each trait individually.

### 4.4. DNA Isolation, Genotyping and Marker Screening

DNA isolation from leaf tissue and genotyping were performed by SGS Institute Fresenius—Trait Genetics Section Gatersleben, Germany. Genotyping was conducted using 19K Brassica SNP microarrays in every year of the experiment.

### 4.5. Genome-Wide Association Studies (GWAS)

For the association analysis, only SNP sequences meeting the following criteria were selected: minor allele frequency (MAF) > 0.25 and the missing observation fractions < 10%. Association mapping was conducted based on lines mean trait values and the marker data, using a mixed linear model (MLM) approach. This model incorporated population structure inferred via eigenanalysis and modeled as random effects [27,51]. All statistical analyses and result visualizations were carried out using GenStat 23.1 software [52], specifically employing the QSASSOCIATION procedure. This procedure implements a mixed-model marker–trait association analysis (also known as linkage disequilibrium mapping) for data obtained from a single-trait trial. To control for false-positive associations due to population structure and relatedness, the model included a genetic relatedness correction. The RELATIONSHIPMODEL = eigenanalysis option was used, which identifies major principal components from the marker matrix to account for population stratification [53]. The scores of the significant principal components were incorporated as covariates in the MLM, providing an approximation of the genetic variance–covariance structure via a kinship matrix. The statistical significance of marker–trait associations was evaluated using *p*-values adjusted for multiple testing using the Benjamini–Hochberg false discovery rate correction method.

### 4.6. GO Enrichment Analyses on Candidate Genes

Gene ontology (GO) analyses of the predicted target genes were conducted using GO (www.geneontology.org (accessed on 5 May 2025)). An analytical tool from the DAVID bioinformatic tools (https://davidbioinformatics.nih.gov/summary.jsp (accessed on 5 May 2025)) was used, which is continuously updated with GO annotations. An in-depth enrichment analysis bubble chart was constructed for visualization by SRPLOT website (www.bioinformatics.com.cn/srplot (accessed on 10 May 2025)).

## 5. Conclusions

This study identified a comprehensive set of SNP markers associated with key seed quality traits in winter oilseed rape, including oil, protein, and fiber fractions (ADF and NDF). From 18,566 detected SNPs, 3782 met stringent criteria and were used for association mapping, resulting in 3189 significant associations across three years. The highest number of associations was observed for protein (3480), followed by NDF (3662), ADF (3422), and oil (2046). Individual markers explained up to 35% of phenotypic variation, indicating strong genetic control of these traits.

Mapping of selected SNPs revealed candidate genes involved in critical biological processes: protein biosynthesis and post-translational modification, lipid metabolism regulated by phosphorylation, and transcriptional control of cell wall polysaccharide synthesis. GO enrichment analysis confirmed that these genes participate in pathways influencing storage protein stability, fatty acid and triacylglycerol biosynthesis, and lignin/cellulose deposition.

These findings provide valuable genomic resources for marker-assisted selection (MAS) and genomic selection (GS) in rapeseed breeding. The identified markers and candidate genes can accelerate the development of cultivars with optimized seed composition—high oil and protein content combined with reduced fiber—addressing the growing demand for high-quality raw materials in food, feed, and biofuel industries. Future work should be focused on the validation of these markers in diverse genetic backgrounds and integrating them into breeding pipelines to enhance efficiency and precision. The SNP markers obtained in our studies may be a valuable tool for further association and marker-assisted selection (MAS) in rapeseed breeding aimed at improving seed quality, especially in terms of protein, oil, and fiber content.

## Figures and Tables

**Table 1:** The number of significant markers for individual traits.

Trait	The Number of Significant Markers				Total Number of Associations
	All	Year			
		2022	2023	2024	
protein	2350	1061	1768	651	3480
oil	1551	829	461	756	2046
ADF	2192	1733	1629	60	3422
NDF	2301	1564	1697	401	3662

**Table 2:** The range (min-max) of the percentages of variation in observed traits explained by individual markers.

Trait	2022	2023	2024
protein	0.9–7.3	0.8–14.1	0.9–25.4
oil	0.8–9.0	0.8–7.0	0.9–35.0
ADF	0.8–10.4	0.8–11.7	0.9–1.9
NDF	0.9–12.5	0.8–10.8	0.9–13.4

**Table 3:** Meteorological conditions in Borowo during the vegetation season of winter oilseed rape in 2021/2022, 2022/2023 and 2023/2024.

Basic Weather Parameters	2021/2022	2022/2023	2023/2024
Mean temperature (°C)			
Annual	9.7	9.26	10.9
Critical season of autumn (Months: September, October and November)	10.1	9.8	11.6
Of the coldest month of winter	−0.1/XII	1.1/XII	0.8/XII
Critical season of spring (Months: April, May, June and July)	14.2	15.1	16.8
Sum of precipitation (mm)			
Critical season of autumn (Months: September, October and November)	95.2	111.9	134.3
Critical season of winter (Months: December, January, February, and March)	101.8	135.4	206
Critical season of spring (Months: April, May, June and July)	93.8	206	273.7

## Data Availability

The original contributions presented in this study are included in the article/Supplementary Materials. Further inquiries can be directed to the corresponding author.

## Supplementary Materials

The following supporting information can be downloaded at: https://www.mdpi.com/article/10.3390/ijms262411931/s1.
