# Supplementary material for: Genome-Wide Association Study for Markers Related to Protein, Fiber (ADF and NDF) and Oil Content in Winter Oilseed Rape Seeds (Brassica napus L.)
Source: Int J Mol Sci. 2025 Dec 11;26(24):11931. doi: 10.3390/ijms262411931 (PMC12732502; doi:10.3390/ijms262411931)
Supplement: Supplementary file 1 [file ijms-26-11931-s001.zip › Supplementary Table S1.pdf]

Table S1. Selected SNP markers associated with a single trait for further analysis with protein, oil, ADF and NDF, based on *p*-values

Lp.	Chrom.	Position	Marker	*p*-value		
		Protein		2022	2023	2024
**1.**	chrA03	36522076	Bn-A01-p22363809	0.0114001384	0.0000001478	0.000331003
**2.**	chrA03	37650693	Bn-Scaffold000096-p760723	0.0114001384	0.0000001478	0.000331003
**3.**	chrA03	38059524	Bn-Scaffold000162-p320522	0.0220066881	0.0000000219	0.001056612
**4.**	chrA06	2450833	Bn-scaff_20866_1-p108216	0.0000520421	0.0000000010	0.003156841
**5.**	chrA07	10544969	Bn-A07-p7311296	0.0004615360	0.0000013586	0.011046795
**6.**	chrA07	16048204	Bn-A07-p11698185	0.0000988452	0.0000000000	0.017484448
**7.**	chrA07	21291972	Bn-A07-p16632905	0.0000583792	0.0000008743	0.006371118
**8.**	chrA07	21392482	Bn-A07-p16716464	0.0000253486	0.0000006748	0.00610437
**9.**	chrA07	21406561	Bn-A07-p16727616	0.0000089064	0.0000002404	0.004989912
**10.**	chrA08	2870583	Bn-scaff_20866_1-p111789	0.0000832743	0.0000000000	0.004664723
**11.**	chrC06	5459303	Bn-scaff_16485_1-p668116	0.0000834848	0.0000000000	0.010794825
**12.**	chrC06	5468706	Bn-scaff_16485_1-p676613	0.0000834848	0.0000000000	0.010794825
		Oil		2022	2023	2024
**13.**	chrA01	21475775	Bn-A01-p20147405	0.0000430066	0.0053302327	0.003621735
**14.**	chrA01	21497021	Bn-A01-p20167230	0.0000430066	0.0053302327	0.003621735
**15.**	chrA01	24566303	Bn-A01-p23330944	0.0000001108	0.0000774856	0.017884532
**16.**	chrA03	6110810	Bn-scaff_18322_1-p2096853	0.0004344912	0.0029678253	0.014313739
**17.**	chrA03	20776695	Bn-scaff_16130_2-p374682	0.0053657427	0.0037940941	0.000207687
**18.**	chrA07	13642452	Bn-scaff_19253_1-p256729	0.0034602314	0.0007815014	0.008485438
**19.**	chrC03	823415	Bn-scaff_26320_1-p298590	0.0001678828	0.0004817189	0.015534853
**20.**	chrC03	11335737	Bn-scaff_18322_1-p2084715	0.0004344912	0.0029678253	0.014313739
**21.**	chrC03	40306111	Bn-scaff_15695_1-p611509	0.0002083266	0.0001955531	0.00713224
**22.**	chrC03	41538617	Bn-scaff_18855_1-p332361	0.0002006504	0.0001691578	0.008566361
**23.**	chrC03	41614157	Bn-scaff_17869_1-p661079	0.0002006504	0.0001691578	0.008566361
**24.**	chrC03	45218965	Bn-scaff_19310_1-p73626	0.0086563849	0.0008486505	0.000511467
**25.**	chrC03	45222126	Bn-scaff_19310_1-p76145	0.0032770571	0.0002622670	7.36845E-05
**26.**	chrC03	45228366	Bn-scaff_19310_1-p86941	0.0032770571	0.0002622670	7.36845E-05
		ADF		2022	2023	2024
**27.**	chrA04	21757646	Bn-A05-p677619	0.0001761288	0.0000065538	0.028552571
**28.**	chrA05	19276	Bn-A05-p474257	0.0000376113	0.0000571184	0.040518217

**29.**	chrA05	500940	Bn-A05-p114598	0.0003938841	0.0000224512	0.013029401
			**NDF**	**2022**	**2023**	**2024**
**30.**	chrA01	7823642	Bn-A01-p7646601	0.0181783115	0.0000000008	0.010630943
**31.**	chrA03	27582636	Bn-A03-p26833555	0.0022206361	0.0026554754	0.017581535
**32.**	chrA03	27582934	Bn-A03-p26833841	0.0019441766	0.0021402559	0.01302347
**33.**	chrA04	2916077	Bn-A04-p2949339	0.0004378453	0.0000021561	0.007194111
**34.**	chrA05	27295530	Bn-A05-p22221563	0.0029364401	0.0000000578	0.006497565
**35.**	chrA06	26102279	Bn-scaff_17291_1-p475978	0.0029761359	0.0027571345	0.010609459
**36.**	chrC02	45541347	Bn-scaff_17623_1-p546659	0.0062204759	0.0077313201	0.014465571
**37.**	chrC03	62129802	Bn-scaff_16755_1-p223829	0.0000183164	0.0000063928	0.022987198
**38.**	chrC04	17479037	Bn-scaff_23907_1-p3780	0.0001019309	0.0002002583	0.014091466
**39.**	chrC07	23978108	Bn-scaff_21711_1-p34999	0.0050049149	0.0012587783	0.001362363
**40.**	chrC07	24299404	Bn-scaff_18520_1-p202169	0.0052168662	0.0029691255	0.010272823
