# Supplementary material for: Genome-Wide Association Study for Markers Related to Protein, Fiber (ADF and NDF) and Oil Content in Winter Oilseed Rape Seeds (Brassica napus L.)
Source: Int J Mol Sci. 2025 Dec 11;26(24):11931. doi: 10.3390/ijms262411931 (PMC12732502; doi:10.3390/ijms262411931)
Supplement: Supplementary file 1 [file ijms-26-11931-s001.zip › Supplementary Table S2.pdf]

Table S2. Selected SNP markers associated with multiple traits simultaneously for further analysis with protein, oil, ADF and NDF, based on *p*-values

Lp.	Chrom.	Position	Marker	*p*-value		
			Protein + NDF	Protein		
				2022	2023	2024
				NDF		
				2022	2023	2024
**41.**	chrA05	9898975	Bn-scaff_17088_2-p126958	0.0011500258	0.0000359811	0.009042514
				0.0000693080	0.0000011134	0.017103
**42.**	chrA05	25791860	Bn-scaff_21369_1-p1167373	0.0005244927	0.0000016298	0.047097766
				0.0001404618	0.0000004590	0.041584
**43.**	chrA06	27089872	Bn-scaff_21711_1-p76752	0.0183308741	0.0000039807	0.00043775
				0.0000810967	0.0001772213	0.005723
**44.**	chrA07	10255486	Bn-A07-p7069624	0.0000439343	0.0000000057	0.012091355
				0.0000000904	0.0000000136	0.003627
**45.**	chrA07	10345055	Bn-A07-p7139702	0.0000149542	0.0000000000	0.011282355
				0.0000000700	0.0000000040	0.007275
**46.**	chrA07	10471098	Bn-A07-p7244763	0.0000149542	0.0000000000	0.011282355
				0.0000000700	0.0000000040	0.007275
**47.**	chrA09	34713840	Bn-A09-p29163307	0.0202902230	0.0000704916	0.000141716
				0.0000935132	0.0001506259	0.010681
**48.**	chrC01	20696433	Bn-scaff_16984_1-p22326	0.0001275679	0.0000000001	0.004540966
				0.0000018093	0.0000049768	0.048377
**49.**	chrC02	16583615	Bn-scaff_16565_1-p1169320	0.0041401953	0.0000079334	0.017277462
				0.0002884220	0.0008401538	0.037642
**50.**	chrC03	1228548	Bn-A07-p11724009	0.0000891608	0.0000000000	0.010569711
				0.0000002901	0.0000010370	0.040702
**51.**	chrC03	29335645	Bn-scaff_17521_1-p3935	0.0000556787	0.0000460287	0.000493953
				0.0000551939	0.0000880263	0.002587
**52.**	chrC03	29338183	Bn-scaff_17521_1-p1390	0.0004880344	0.0000319444	0.001965593
				0.0005496973	0.0011375399	0.007278
**53.**	chrC03	29339676	Bn-scaff_28562_1-p33404	0.0000556787	0.0000460287	0.000493953
				0.0000551939	0.0000880263	0.002587
**54.**	chrC03	59999793	Bn-scaff_16148_1-p294989	0.0003983632	0.0000005587	0.002807829
				0.0001413208	0.0008353279	0.00955
**55.**	chrC03	60098923	Bn-scaff_16148_1-p159973	0.0003309486	0.0000268609	0.004284011
				0.0000686881	0.0034045274	0.016968

**56.**	chrC04	17478619	Bn-scaff_23907_1-p4193	0.0002018336	0.0001614392	0.027118011
				0.0000094753	0.0000691569	0.005604
**57.**	chrC05	5239517	Bn-scaff_21496_1-p440091	0.0007603368	0.0000000005	0.001874039
				0.0001536863	0.0000012237	0.003545
**58.**	chrC05	5245222	Bn-scaff_21496_1-p442411	0.0001771956	0.0000000585	0.00583008
				0.0000228322	0.0003347682	0.006119
**59.**	chrC05	5646545	Bn-scaff_15712_10-p380937	0.0076885665	0.0000000068	0.010326703
				0.0057774339	0.0000145557	0.023164
**60.**	chrC06	15966905	Bn-scaff_18002_1-p29854	0.0097595173	0.0000155827	0.006967311
				0.0012006154	0.0000002681	0.00532
**61.**	chrC06	16700531	Bn-scaff_17088_2-p166858	0.0011500258	0.0000359811	0.009042514
				0.0000693080	0.0000011134	0.017103
**62.**	chrC06	16701621	Bn-scaff_17088_2-p165768	0.0057437186	0.0002153988	0.003984964
				0.0004368603	0.0000094245	0.005235
**63.**	chrC06	17054266	Bn-scaff_18439_1-p277935	0.0024306562	0.0001335537	0.0034871
				0.0002553388	0.0000130407	0.006403
**64.**	chrC06	17106308	Bn-scaff_18439_1-p315543	0.0031883610	0.0000142215	0.001426209
				0.0003058822	0.0000029338	0.001635
**65.**	chrC06	20456436	Bn-scaff_16903_1-p230137	0.0396110739	0.0000245997	0.000256496
				0.0074603562	0.0000019037	0.000805
**66.**	chrC06	21079000	Bn-scaff_15818_1-p292872	0.0396110739	0.0000245997	0.000256496
				0.0074603562	0.0000019037	0.000805
**67.**	chrC07	319860	Bn-A07-p7118584	0.0000560395	0.0000000027	0.005304954
				0.0000004620	0.0000000384	0.003923
**68.**	chrC07	23289962	Bn-scaff_17972_1-p361917	0.0082071169	0.0000000837	0.001315989
				0.0000072482	0.0000023394	0.013426
**69.**	chrC07	24208489	Bn-scaff_18520_1-p117612	0.0310914489	0.0016892134	0.022569116
				0.0010636902	0.0006708653	0.013173
			**ADF + NDF**		**ADF**	
				**2022**	**2023**	**2024**
					**NDF**	
				**2022**	**2023**	**2024**
**70.**	chrA04	14373387	Bn-A04-p11453319	0.0000957836	0.0000000373	0.023161929
				0.0025721140	0.0000000075	0.018792
**71.**	chrA04	14383873	Bn-A04-p11463316	0.0006752290	0.0000000738	0.017063718
				0.0083988162	0.0000000046	0.00334
**72.**	chrA04	16103874	Bn-A04-	0.0428623670	0.0000058419	0.02730438

			p13461501	0.0378435084	0.0000090683	0.020305
**73.**	chrA04	16348487	Bn-A04-p13705760	0.0015853372	0.0000002318	0.014145838
				0.0013934308	0.0000054262	0.00818
**74.**	chrA04	16372285	Bn-A04-p13726056	0.0004973157	0.0000004557	0.006559347
				0.0008771518	0.0003227525	0.007629
**75.**	chrA04	16372489	Bn-scaff_16517_1-p354305	0.0002359644	0.0000000199	0.012421174
				0.0003084338	0.0000018012	0.005875
**76.**	chrC07	459283	Bn-scaff_17326_1-p536514	0.0002503707	0.0000035031	0.008681266
				0.0000325661	0.0000543870	0.017962
			**Protein + Oil**	**Protein**		
				**2022**	**2023**	**2024**
				**Oil**		
				**2022**	**2023**	**2024**
**77.**	chrA05	26956534	Bn-scaff_17441_1-p573618	0.0000035260	0.0008208443	0.009835466
				0.0000017165	0.0040696659	0.014642
**78.**	chrC03	44862419	Bn-scaff_18356_1-p272201	0.0221466345	0.0000003293	0.000463191
				0.0303912742	0.0109344559	0.003327
			**Protein + ADF + NDF**	**Protein**		
				**2022**	**2023**	**2024**
				**ADF**		
				**2022**	**2023**	**2024**
				**NDF**		
				**2022**	**2023**	**2024**
**79.**	chrA04	14369542	Bn-A04-p11449464	0.0186581735	0.0000000086	0.041683792
				0.0009363388	0.0000001109	0.018235954
				0.0105035847	0.0000000124	0.004061
**80.**	chrA08	487462	Bn-scaff_16755_1-p436280	0.0044097750	0.0000000042	0.003013703
				0.0000327637	0.0000233288	0.021611594
				0.0000111665	0.0000004864	0.000573
**81.**	chrC05	5003989	Bn-scaff_21496_1-p191960	0.0360330804	0.0000000003	0.005130144
				0.0020050647	0.0000008010	0.048687769
				0.0151274901	0.0000006187	0.007538
