# Supplementary material for: Genome-Wide Association Study for Markers Related to Protein, Fiber (ADF and NDF) and Oil Content in Winter Oilseed Rape Seeds (Brassica napus L.)
Source: Int J Mol Sci. 2025 Dec 11;26(24):11931. doi: 10.3390/ijms262411931 (PMC12732502; doi:10.3390/ijms262411931)
Supplement: Supplementary file 1 [file ijms-26-11931-s001.zip › Supplementary Table S3.pdf]

Table S3. Genes located close to selected SNP markers, with their position and length specified.

Marker	Chrom.	Localization	Candidate Genes	Length [bp]
			**Protein**	
**Bn-A01-p22363809**	A03	36516933 - 36517261	*Brassica napus* F-box protein PP2-B1 (LOC106396140)	329
	A03	36532464 - 36533294	*Brassica rapa* GLABROUS1 enhancer-binding protein (LOC103838558)	831
	A03	36489122 - 36489712	*Brassica napus* uncharacterized (LOC106355679)	591
**Bn-Scaffold000096-p760723**	A03	37642109 - 37642324	*Brassica napus* retrotransposon-like protein 1 (LOC106404241)	216
	A03	37584126 - 37585501	*Brassica napus* rho GDP-dissociation inhibitor 1 (LOC106395781)	1376
	A03	37747105 - 37747518	*Brassica napus* 14 kDa proline-rich protein DC2.15 (LOC106411407)	414
	A03	37757583 - 37758059	*Brassica napus* uncharacterized (LOC106411406)	477
**Bn-Scaffold000162-p320522**	A03	38042196 - 38042794	*Brassica napus* mitochondrial import inner membrane translocase subunit TIM9 (LOC106391035)	599
	A03	38105556 - 38111255	*Brassica napus* DNA-directed RNA polymerase IV subunit 1 (LOC106406243)	5700
	A03	37988851 - 37989345	*Brassica rapa* ervatamin-B-like (LOC103847912)	495
	A03	38167588 - 38168217	*Brassica napus* uncharacterized (LOC106418873)	630
**Bn-scaff_20866_1-p108216**	A06	2450796 - 2451781	*Brassica napus* uncharacterized (LOC125610139)	986
	A06	2454954 - 2455874	*Brassica napus* agamous-like MADS-box protein AGL93 (LOC125610138)	921
	A06	2458356 - 2459495	*Brassica napus* uncharacterized (LOC125588690)	1140
**Bn-A07-p7311296**	A07	10545262 - 10547188	*Brassica napus* IST1-like protein (LOC106367562)	1927
	A07	10550520 - 10550861	*Brassica napus* uncharacterized (LOC106414078)	342
	A07	10539707 - 10540564	*Brassica napus* uncharacterized (BNAANNG41370D)	858
	A07	10533281 - 10535378	*Brassica napus* LEAF RUST 10 DISEASE-RESISTANCE LOCUS RECEPTOR-LIKE PROTEIN KINASE-like 1.1 (LOC106412535)	2098
**Bn-A07-p11698185**	A07	16047308 - 16048248	*Brassica rapa* 50S ribosomal protein L17, chloroplastic (LOC103829970)	941
	A07	:16045690 - 16046367	*Raphanus sativus* NDR1/HIN1-like protein 6 (LOC108840889)	678
	A07	16050162 - 16051792	*Brassica napus* vesicle-associated membrane protein 727 (LOC106352858)	1631

	A07	16041391 - 16043789	*Brassica napus* FKBP12-interacting protein of 37 kDa (LOC106407332)	2399
	A07	16031456 - 16036715	*Brassica napus* uncharacterized (LOC125589865)	5260
	A07	16024270 - 16025875	*Brassica rapa* FKBP12-interacting protein of 37 kDa (LOC103829967)	1606
**Bn-A07-p16632905**	A07	21295637 - 21297081	*Brassica napus* uncharacterized (LOC106378797)	1445
	A07	21300702 - 21302086	*Brassica napus* histidine-rich glycoprotein (LOC106378957)	1385
	A07	21282199 - 21284458	*Brassica napus* BTB/POZ domain-containing protein At1g67900 (LOC106378554)	2260
**Bn-A07-p16716464**	A07	21389881 - 21392352	*Brassica napus* uncharacterized (BNAA07G24940D)	2472
	A07	21395582 - 21400419	*Brassica napus* lipoxygenase 6, chloroplastic-like (LOC106410673)	4838
	A07	21384001 - 21386667	*Brassica napus* signal recognition particle subunit SRP72-like (LOC106436808)	2667
	A07	21379411 - 21383706	*Brassica napus* probable thimet oligopeptidase (LOC106410390)	4296
	A07	21377898 - 21378940	*Brassica napus* protein HHL1, chloroplastic-like (LOC106410393)	1043
	A07	21375417 - 21377254	*Brassica napus* two-component response regulator ARR11-like (LOC125576273)	1838
**Bn-A07-p16727616**	A07	21413176 - 21416991	*Brassica napus* E3 ubiquitin-protein ligase ORTHRUS 2 (LOC106411162)	3816
	A07	21418215 - 21426279	*Brassica napus* DNA polymerase zeta catalytic subunit-like (LOC106410865)	8065
	A07	21428473 - 21434166	*Brassica napus* mannosyl-oligosaccharide glucosidase GCS1 (LOC125588628)	5694
**Bn-scaff_20866_1-p111789**	A08	2870088 - 2872966	*Brassica napus* uncharacterized LOC106423775	2879
**Bn-scaff_16485_1-p668116**	A08	2876747 - 2881222	*Brassica napus* nucleolin 1-like (LOC106423765)	4476
**Bn-scaff_16485_1-p676613**	A08	2858416 - 2861108	*Brassica napus* translation initiation factor eIF-2B subunit delta-like (LOC106360503)	2693
			**Oil**	
**Bn-A01-p20147405**	A01	21462950 - 21465778	*Brassica napus* protein tesmin/TSO1-like CXC 3 (LOC106454039)	2829
	A01	21498719 - 21500414	*Brassica napus* serine/threonine-protein kinase STY13 (LOC106454026)	1696
	A01	21501058 - 21501644	*Brassica napus* transcription repressor MYB5-like (LOC106444546)	587
	A01	21502427 - 21503278	*Brassica napus* probable rRNA-processing protein EBP2 homolog (LOC106454016)	852
	A01	21458892 - 21459272	*Brassica napus* F-box protein DOR-like (LOC106454067)	381

	A01	21451013 - 21456307	*Brassica napus* protein NETWORKED 1A-like (LOC106454063)	5295
**Bn-A01-p20167230**	A01	21498719 - 21500414	*Brassica napus* serine/threonine-protein kinase STY13 (LOC106454026)	1696
	A01	21501058 - 21501644	*Brassica napus* transcription repressor MYB5-like (LOC106444546)	587
	A01	21502427 - 21503278	*Brassica napus* probable rRNA-processing protein EBP2 homolog (LOC106454016)	852
	A01	21509871 - 21510398	*Brassica napus* RING-H2 finger protein ATL16-like (LOC106365208)	528
**Bn-A01-p23330944**	A01	24564433 - 24566479	*Brassica napus* ubiquitin-conjugating enzyme E2 32-like (LOC125577366)	2047
	A01	24562717 - 24563699	*Brassica napus* universal stress protein PHOS34 (LOC106396771)	983
	A01	24558540 - 24562012	*Brassica napus* uncharacterized (LOC125577361)	3473
	A01	24571899 - 24576654	*Brassica napus* glutathione S-transferase T3-like (LOC111200511)	4756
**Bn-scaff_18322_1-p2096853**	A03	6104744 - 6106443	*Brassica napus* WD repeat-containing protein 25-like (LOC106443326)	1700
	A03	6120417 - 6122593	*Brassica napus* indole-3-acetic acid-amido synthetase GH3.6-like (LOC106437258)	2177
	A03	6124277 - 6126019	*Brassica napus* NAD(P)H dehydrogenase (quinone) FQR1 (LOC106437251)	1743
	A03	6135224 - 6136240	*Brassica napus* serine/threonine-protein kinase ZRK1-like (LOC106441621)	1017
	A03	6137782 - 6140295	*Brassica napus* receptor-like protein kinase THESEUS 1 (LOC106437235)	2514
**Bn-scaff_16130_2-p374682**	A03	20775417 - 20778570	*Brassica napus* probable disease resistance protein At1g59620 (LOC111214216)	3154
	A03	20781368 - 20783264	*Brassica napus* 3',5'-nucleoside bisphosphate phosphatase (LOC106439749)	1897
	A03	20784263 - 20785048	*Brassica napus* c-Myc-binding protein-like (LOC125576143)	786
	A03	20771044 - 20772494	*Brassica napus* non-specific lipid transfer protein GPI-anchored 11-like (LOC125607062)	1451
	A03	20765131 - 20768010	*Brassica napus* somatic embryogenesis receptor kinase 4-like (LOC106439751)	2880
**Bn-scaff_19253_1-p256729**	A07	13643832 - 13646228	*Brassica napus* pentatricopeptide repeat-containing protein At2g26790, mitochondrial (LOC106358457)	2397
	A07	13634294 - 13637021	*Brassica napus* stomatal closure-related actin-binding protein 1 (LOC106358458)	2728
	A07	13624729 - 13627500	*Brassica napus* probable inactive receptor kinase At2g26730 (LOC106358459)	2772
	A07	13657759 - 13659932	*Brassica napus* hydroxymethylglutaryl-CoA lyase, mitochondrial (LOC106358455)	2174
	A07	13660476 -	*Brassica napus* dnaJ homolog subfamily C	10567

		13671042	GRV2 (LOC106358454)	
	A07	13672716 - 13674797	*Brassica napus* 4-diphosphocytidyl-2-C-methyl-D-erythritol kinase, chloroplastic (LOC106358453)	2082
**Bn-scaff_26320_1-p298590**	C03	823030 - 824530	*Brassica rapa* F-box/LRR-repeat protein At3g60040-like (LOC103842341)	1501
	C03	822366 - 822838	*Brassica rapa* pathogenesis-related protein 1 (LOC103862259)	473
	C03	826010 - 826294	*Brassica napus* uncharacterized (LOC106397948)	285
	C03	829806 - 830768	*Brassica napus* nicotianamine synthase 1 (LOC106428166)	963
**Bn-scaff_18322_1-p2084715**	C03	11336425 - 11338534	*Brassica napus* indole-3-acetic acid-amido synthetase GH3.6 (LOC106346022)	2110
	C03	11342513 - 11343203	*Camelina sativa* tyrosyl-DNA phosphodiesterase 1 (LOC104769757)	691
	C03	11343330 - 11344783	*Brassica napus* NAD(P)H dehydrogenase (quinone) FQR1-like (LOC106346023)	1454
**Bn-scaff_15695_1-p611509**	C03	40305295 - 40306969	*Brassica napus* zyxin-like (LOC111201546)	1675
	C03	40302351 - 40304538	*Brassica napus* classical arabinogalactan protein 9 (LOC106416322)	2188
	C03	40315200 - 40316306	*Brassica napus* uncharacterized (LOC125588786)	1107
**Bn-scaff_18855_1-p332361**	C03	41538515 - 41541116	*Brassica napus* tetracycline resistance protein, class E (LOC106389468)	2602
	C03	41530077 - 41530857	*Brassica napus* tetracycline resistance protein, class E (LOC106389468)	781
	C03	41557586 - 41558864	*Brassica napus* uncharacterized (LOC106369409)	1279
	C03	41518224 - 41521204	*Brassica napus* uncharacterized (LOC125590487)	2981
**Bn-scaff_17869_1-p661079**	C03	41603953 - 41604726	*Brassica napus* uncharacterized (LOC125583733)	774
	C03	41621949 - 41622257	*Brassica napus* B3 domain-containing protein At1g08985-like (LOC125583734)	309
	C03	41602095 - 41602886	*Brassica napus* uncharacterized (LOC111203944)	792
	C03	41598143 - 41599152	*Brassica napus* NAC domain-containing protein 6 (LOC111204246)	1010
**Bn-scaff_19310_1-p73626**	C03	45208994 - 45215488	*Brassica napus* protein CHROMATIN REMODELING 24 (LOC106348031)	6495
**Bn-scaff_19310_1-**	C03	45226388 - 45226915	*Brassica napus* pectinesterase inhibitor 12-like (LOC106348047)	528

**p76145**				
**Bn-scaff_19310_1-p86941**	C03	45228392 - 45228859	*Brassica oleracea* var. *oleracea* uncharacterized LOC106326523	468
	C03	45231050 - 45233937	*Brassica napus* protein kinase STUNTED (LOC106367343)	2888
	C03	45197089 - 45204107	*Brassica napus* DNA polymerase delta catalytic subunit (LOC106348005)	7019
	C03	45197089 - 45204107	*Brassica napus* farnesylcysteine lyase (LOC106348069)	7019
			**ADF**	
**Bn-A05-p677619**	A04	21757013 - 21758454	*Brassica napus* transcription factor ABA-INDUCIBLE bHLH-TYPE (LOC106453228)	1442
	A04	21759756 - 21761491	*Brassica napus* phosphatidylinositol 4-kinase gamma 4-like (LOC106453226)	1736
	A04	21762400 - 21763863	*Brassica napus* putative RING-H2 finger protein ATL21A (LOC125608392)	1464
	A04	21764900 - 21766117	*Brassica napus* putative RING-H2 finger protein ATL21B (LOC106345696)	1218
	A04	21768152 - 21768924	*Brassica napus* transcription factor CPC-like (LOC106345694)	773
**Bn-A05-p474257**	A05	17362 - 18529	*Brassica napus* protein CURVATURE THYLAKOID 1B, chloroplastic (LOC106415795)	1168
	A05	19978 - 24683	*Brassica napus* protein CCA1 (LOC106450471)	4706
	A05	14445 - 15797	*Brassica napus* transcription factor bHLH70-like (LOC106450473)	1353
**Bn-A05-p114598**	A05	499353 - 504712	*Brassica napus* DExH-box ATP-dependent RNA helicase DExH8 (LOC106450420)	5360
	A05	506288 - 508215	*Brassica napus* E3 ubiquitin-protein ligase RFI2 (LOC106450417)	1928
	A05	505066 - 505792	*Brassica rapa* NADH dehydrogenase [ubiquinone] iron-sulfur protein 5-B (LOC103866353)	727
	A05	496878 - 497498	*Brassica napus* pectinesterase inhibitor 6-like (LOC106450421)	621
	A05	508756 - 509675	*Brassica napus* universal stress protein PHOS34 (LOC106450418)	920
			**NDF**	
**Bn-A01-p7646601**	A01	7819698 - 7824322	*Brassica napus* serine/threonine-protein kinase fray2 (LOC106415334)	4625
	A01	7824956 - 7825699	*Brassica napus* uncharacterized (LOC106415336)	744
	A01	7817610 - 7819146	*Brassica napus* uncharacterized (LOC106415335)	1537
	A01	7816145 - 7817189	*Brassica napus* 2-keto-3-deoxy-L-rhamnonate aldolase-like (LOC106413970)	1045
	A01	7813282 - 7814601	*Brassica napus* 2-keto-3-deoxy-L-rhamnonate aldolase-like (LOC106413150)	1320

	A01	7830781 - 7833276	*Brassica napus* metal-nicotianamine transporter YSL1-like (LOC106415424)	2496
**Bn-A03-p26833555**	A03	27582661 - 27582930	Brassica rapa wound-responsive protein-related (LOC103861817)	270
**Bn-A03-p26833841**	A03	27579012 - 27581926	*Brassica napus* external alternative NAD(P)H-ubiquinone oxidoreductase B1, mitochondrial (LOC111214317)	2915
	A03	27576977 - 27578304	*Brassica napus* uncharacterized (LOC111198034)	1328
	A03	27574364 - 27575993	*Brassica napus* protein ULTRAPETALA 1-like (LOC111214316)	1630
	A03	27594107 - 27595497	*Brassica napus* expansin-B3-like (LOC106392800)	1391
	A03	27597179 - 27597730	*Brassica rapa* thioredoxin O2, mitochondrial (LOC103840275)	552
**Bn-A04-p2949339**	A04	2917796 - 2920156	*Brassica napus* ABC transporter G family member 19-like (LOC106445340)	2361
	A04	2909325 - 2910827	*Brassica napus* probable pectate lyase 4 (LOC106445342)	1503
	A04	2907211 - 2909112	*Brassica napus* exocyst complex component EXO70H1 (LOC106445341)	1902
	A04	2895739 - 2898320	*Brassica napus* caffeoylshikimate esterase-like (LOC106447932)	2582
**Bn-A05-p22221563**	A05	27294900 - 27296321	*Brassica napus* ras-related protein RABE1e (LOC111215768)	1422
	A05	27293082 - 27294476	*Brassica napus* ras-related protein RABC2b (LOC106452778)	1395
	A05	27297142 - 27298198	*Brassica napus* ankyrin repeat domain-containing protein 1 (LOC106454282)	1057
	A05	27299754 - 27301915	*Brassica rapa* serine/threonine protein phosphatase 2A 57 kDa regulatory subunit B' beta isoform (LOC103870509)	2162
	A05	27288855 - 27292474	*Brassica napus* phosphatidylinositol 4-phosphate 5-kinase 9 (LOC111197836)	3620
**Bn-scaff_17291_1-p475978**	A06	26100662 - 26103435	*Brassica napus* GDP-mannose 3,5-epimerase (LOC106348783)	2774
	A06	26108092 - 26111150	*Brassica rapa* serine/threonine protein phosphatase 2A regulatory subunit B''beta (LOC103874595)	3059
	A06	26095257 - 26097033	*Brassica rapa* basic leucine zipper 63 (LOC103874593)	1777
	A06	26093354 - 26094370	*Brassica napus* sec-independent protein translocase protein TATA, chloroplastic (LOC106348781)	1017
	A06	26113026 - 26115049	*Brassica napus* uncharacterized (LOC106348792)	2024
**Bn-scaff_17623_1-p546659**	C02	45541214 - 45543812	*Brassica napus* putative cysteine-rich receptor-like protein kinase 31 (LOC111206366)	2599

	C02	45536354 - 45537659	*Brassica napus* putative H/ACA ribonucleoprotein complex subunit 1-like protein 1 (LOC125581870)	1306
	C02	45533601 - 45534024	*Brassica rapa* putative nuclease HARBI1 (LOC103854825)	424
	C02	45530893 - 45532731	*Brassica napus* small polypeptide DEVIL 19 (LOC106444086)	1839
	C02	45550640 - 45554667	*Brassica napus* calmodulin-binding protein 60 G (LOC111198122)	4028
**Bn-scaff_16755_1-p223829**	C03	62127147 - 62129578	*Brassica napus* uncharacterized WD repeat-containing protein C2A9.03-like (LOC106416657)	2432
	C03	62125836 - 62126321	*Brassica napus* photosystem I reaction center subunit V, chloroplastic (LOC106418443)	486
	C03	62133538 - 62136610	*Brassica napus* phosphatidylinositol/phosphatidylcholine transfer protein SFH13 (LOC106418580)	3073
	C03	62122734 - 62123439	*Brassica napus* nucleoside diphosphate kinase 1 (LOC106418549)	706
	C03	62112617 - 62116333	*Brassica napus* chloride channel protein CLC-f (LOC106418676)	3717
	C03	62104627 - 62108124	*Brassica napus* serine/threonine-protein kinase BRI1-like 1 (LOC106416582)	3498
**Bn-scaff_23907_1-p3780**	C04	17474711 - 17474971	*Brassica napus* precursor of CEP4 (LOC106395954)	261
	C04	17485707 - 17487548	*Brassica napus* rop guanine nucleotide exchange factor 14-like (LOC106394390)	1842
	C04	17488393 - 17489920	*Brassica napus* probable aspartic protease At2g35615 (LOC106395991)	1528
	C04	17490041 - 17493354	*Brassica napus* LRR receptor-like serine/threonine-protein kinase FEI 2 (LOC106396620)	3314
**Bn-scaff_21711_1-p34999**	C07	23977220 - 23978302	*Brassica napus* histidine-containing phosphotransfer protein 2 (LOC106410262)	1083
	C07	23975225 - 23976478	*Brassica napus* uncharacterized (LOC106410261)	1254
	C07	23970528 - 23975022	*Brassica napus* alpha-glucan phosphorylase 1 (LOC106410260)	4495
	C07	23984244 - 23985194	*Brassica napus* UDP-glucose 6-dehydrogenase 2 (LOC106409660)	951
**Bn-scaff_18520_1-p202169**	C07	24293859 - 24296953	*Brassica napus* O-fucosyltransferase 27 (BNAC07G49130D)	3095
	C07	24302499 - 24305413	*Brassica napus* WAT1-related protein At3g30340 (LOC106409977)	2915
	C07	24288910 - 24293093	*Brassica napus* AUGMIN subunit 5 (LOC106410472)	4184
	C07	24306179 - 24306861	*Brassica napus* WAT1-related protein At3g30340 (LOC106352325)	683

	C07	24310761 - 24312618	*Brassica napus* alpha/beta hydrolase domain-containing protein 17B (LOC106410274)	1858
	C07	24313062 - 24315000	*Brassica napus* amino acid transporter AVT6A (LOC106410273)	1939
			**Protein + NDF**	
**Bn-scaff_17088_2-p126958**	A05	9904473 - 9904736	*Brassica napus* uncharacterized (LOC111206417)	264
	A05	9908558 - 9909320	*Brassica napus* glutathione S-transferase U28 (LOC125597623)	763
	A05	9915326 - 9917117	*Brassica napus* polyadenylate-binding protein-interacting protein 8 (LOC106442683)	1792
	A05	9917936 - 9918788	*Brassica rapa* retrovirus-related Pol polyprotein from transposon TNT 1-94 (LOC103868417)	853
**Bn-scaff_21369_1-p1167373**	A05	25790817 - 25792797	*Brassica napus* exocyst complex component EXO70A1-like (LOC111215719)	1981
	A05	25793212 - 25794353	*Brassica napus* sm-like protein LSM1B (LOC111215720)	1142
	A05	25787722 - 25789241	*Brassica napus* protein FLUORESCENT IN BLUE LIGHT, chloroplastic-like (LOC111215718)	1520
	A05	25778755 - 25787281	*Brassica napus* putative F-box protein At3g23260 (LOC106378695)	8527
	A05	25799361 - 25801289	*Brassica napus* cation/calcium exchanger 3-like (LOC111215721)	1929
	A05	25801612 - 25803942	*Brassica napus* subtilisin-like protease SBT1.4 (LOC106452253)	2331
**Bn-scaff_21711_1-p76752**	A06	27089452 - 27091289	*Brassica napus* uncharacterized (LOC106349130)	1838
	A06	27087558 - 27088982	*Brassica napus* chorismate mutase 1, chloroplastic (LOC106349127)	1425
	A06	27091714 - 27093967	*Brassica napus* uncharacterized (BNAA06G31010D)	2254
	A06	27095629 - 27096414	*Brassica napus* transmembrane protein 230 (LOC106349131)	786
	A06	27082354 - 27084420	*Brassica napus* pentatricopeptide repeat-containing protein At3g29230 (LOC106349126)	2067
	A06	27099229 - 27101676	*Brassica napus* SNF1-related protein kinase catalytic subunit alpha KIN11-like (LOC106349132)	2448
**Bn-A07-p7069624**	A07	10255150 - 10257601	*Brassica napus* U-box domain-containing protein 6-like (LOC106425390)	2452
	A07	10247808 - 10252496	*Brassica napus* alpha-glucosidase 2 (LOC106356351)	4689
	A07	10244737 - 10246296	*Brassica napus* ATPase WRNIP1-like (LOC106358082)	1560
	A07	10241702 - 10244489	*Brassica napus* glucose-6-phosphate 1-dehydrogenase 3, chloroplastic	2788

			(LOC106358081)	
	A07	10267997 - 10270934	*Brassica rapa* 2,4-dichlorophenol 6-monooxygenase (LOC103829086)	2938
**Bn-A07-p7139702**	A07	10341692 - 10342964	*Brassica napus* uncharacterized (LOC125610307)	1273
	A07	10350765 - 10353059	*Brassica napus* uncharacterized (LOC106373270)	2295
	A07	10334294 - 10335890	*Brassica napus* protein JINGUBANG-like (LOC106358068)	1597
**Bn-A07-p7244763**	A07	10463280 - 10463795	*Brassica napus* uncharacterized (LOC106414470)	516
	A07	10477525 - 10480165	*Brassica napus* WAT1-related protein At1g25270-like (LOC111213908)	2641
	A07	10461337 - 10462812	*Brassica napus* mRNA turnover protein 4 homolog (LOC106412206)	1476
	A07	10459747 - 10460886	*Brassica napus* putative clathrin assembly protein At1g25240 (LOC106414713)	1140
**Bn-A09-p29163307**	A09	34711737 - 34714663	*Brassica napus* pentatricopeptide repeat-containing protein At3g59040 (LOC106368314)	2927
	A09	34716501 - 34718253	*Brassica napus* transcription factor PIF5-like (LOC111200351)	1753
	A09	34722423 - 34723718	*Brassica napus* cytochrome b561 and DOMON domain-containing protein At5g48750-like (LOC125578482)	1296
	A09	34700572 - 34703646	*Brassica napus* probable pectinesterase/pectinesterase inhibitor 35 (LOC106390203)	3075
	A09	34695745 - 34697551	*Brassica napus* magnesium transporter MRS2-4 (LOC106396564)	1807
**Bn-scaff_16984_1-p22326**	C01	20723036 - 20724435	*Brassica napus* GDSL esterase/lipase At5g37690 (LOC106449459)	1400
	C01	20732022 - 20733167	*Brassica napus* LOB domain-containing protein 27-like (LOC106374662)	1146
	C01	20610756 - 20611184	*Brassica napus* uncharacterized (LOC106432086)	429
**Bn-scaff_16565_1-p1169320**	C02	16580816 - 16583387	*Brassica napus* uncharacterized (LOC106381071)	2572
	C02	16579933 - 16580655	*Brassica napus* LEAF RUST 10 DISEASE-RESISTANCE LOCUS RECEPTOR-LIKE PROTEIN KINASE-like 2.7 (LOC106381073)	723
	C02	16576735 - 16578803	*Brassica napus* LEAF RUST 10 DISEASE-RESISTANCE LOCUS RECEPTOR-LIKE PROTEIN KINASE-like 2.7 (LOC106381073)	2069
	C02	16558246 - 16561670	*Brassica napus* glycerophosphodiester phosphodiesterase GDPDL3 (LOC106435324)	3425
**Bn-A07-p11724009**	C03	1235523 - 1236402	*Brassica napus* high mobility group B protein 4-like (LOC111215313)	880
	C03	1237364 -	*Brassica napus* dehydrodolichyl diphosphate	816

		1238179	synthase 6 (LOC106394802)	
	C03	1215104 - 1217957	*Brassica napus* uncharacterized (LOC125574802)	2854
**Bn-scaff_17521_1-p3935**	C03	29329830 - 29333579	*Brassica napus* alpha-L-arabinofuranosidase 1 (LOC106389072)	3750
**Bn-scaff_17521_1-p1390**	C03	29343522 - 29344958	*Brassica napus* uncharacterized (LOC125593448)	1437
	C03	29326594 - 29329206	*Brassica napus* putative pectinesterase/pectinesterase inhibitor 24 (LOC106386342)	2613
	C03	29318930 - 29324922	*Brassica napus* DNA gyrase subunit A, chloroplastic/mitochondrial (LOC106389076)	5993
**Bn-scaff_28562_1-p33404**	C03	29343522 - 29344958	*Brassica napus* uncharacterized (LOC125593448)	1437
	C03	29329830 - 29333579	*Brassica napus* alpha-L-arabinofuranosidase 1 (LOC106389072)	3750
	C03	29326594 - 29329206	*Brassica napus* putative pectinesterase/pectinesterase inhibitor 24 (LOC106386342)	2613
	C03	29318930 - 29324922	*Brassica napus* DNA gyrase subunit A, chloroplastic/mitochondrial (LOC106389076)	5993
**Bn-scaff_16148_1-p294989**	C03	59998823 - 60000248	*Brassica napus* auxin-responsive protein IAA18-like (LOC106427437)	1426
	C03	60001879 - 60002962	*Brassica napus* protein IQ-DOMAIN 27-like (LOC106427477)	1084
	C03	59996634 - 59997787	*Brassica napus* uncharacterized (LOC125583514)	1154
	C03	60011790 - 60014961	*Brassica napus* probable mitochondrial-processing peptidase subunit alpha-1, mitochondrial (LOC106428601)	3172
**Bn-scaff_16148_1-p159973**	C03	60102421 - 60105042	*Brassica napus* protein CHUP1, chloroplastic (LOC106428584)	2622
	C03	60105914 - 60108120	*Brassica napus* aspartic proteinase oryzasin-1 (LOC106351717)	2207
	C03	60115823 - 60117466	*Brassica napus* uncharacterized (LOC106351311)	1644
	C03	60076399 - 60080318	*Brassica napus* myrosinase-binding protein 2-like (LOC125583515)	3920
**Bn-scaff_23907_1-p4193**	C04	17474711 - 17474971	*Brassica napus* precursor of CEP4 (LOC106395954)	261
	C04	17485707 - 17487548	*Brassica napus* rop guanine nucleotide exchange factor 14-like (LOC106394390)	1842
	C04	17488393 - 17489920	*Brassica napus* probable aspartic protease At2g35615 (LOC106395991)	1528

	C04	17490041 - 17493354	*Brassica napus* LRR receptor-like serine/threonine-protein kinase FEI 2 (LOC106396620)	3314
**Bn-scaff_21496_1-p440091**	C05	5236545 - 5240585	*Brassica napus* glycosyl hydrolase 5 family protein (LOC106399587)	4041
**Bn-scaff_21496_1-p442411**	C05	5234486 - 5236296	*Brassica napus* cytochrome P450 86B1-like (LOC106398387)	1811
	C05	5229013 - 5232143	*Brassica napus* oxysterol-binding protein-related protein 1D (LOC106346445)	3131
	C05	5223518 - 5225746	*Brassica napus* actin-related protein 3 (LOC106397187)	2229
	C05	5220676 - 5222406	*Brassica napus* heterogeneous nuclear ribonucleoprotein A1-like (LOC106400908)	1731
**Bn-scaff_15712_10-p380937**	C05	5646851 - 5648931	*Brassica napus* IRK-interacting protein-like (LOC106445591)	2081
	C05	5640831 - 5645232	*Brassica napus* SNARE-interacting protein KEULE (LOC106445588)	4402
	C05	5655638 - 5656678	*Brassica napus* uncharacterized (BNAA06G08230D)	1041
	C05	5616089 - 5618377	*Brassica napus* uncharacterized (BNAC05G09450D)	2289
**Bn-scaff_18002_1-p29854**	C06	15959307 - 15962060	*Brassica napus* nuclear/nucleolar GTPase 2 (LOC106425667)	2754
	C06	15945852 - 15946261	*Brassica napus* uncharacterized (LOC106446734)	410
	C06	15941918 - 15942619	*Brassica napus* TIR-only protein-like (LOC106450380)	702
	C06	15940019 - 15940490	*Brassica napus* caffeoylshikimate esterase-like (LOC106405424)	472
**Bn-scaff_17088_2-p166858**	C06	16703160 - 16704307	*Brassica napus* signal peptidase complex catalytic subunit SEC11C-like (LOC106402543)	1148
	C06	16693660 - 16694004	*Brassica napus* uncharacterized LOC106394856 (LOC106394856)	345
	C06	16711091 - 16712664	*Brassica napus* serine/threonine-protein kinase WAG1 (LOC106431459)	1574
	C06	16680693 - 16683153	*Brassica napus* mitogen-activated protein kinase 18 (LOC111208739)	2461
**Bn-scaff_18439_1-p277935**	C06	17051583 - 17058047	*Brassica napus* 26S proteasome regulatory subunit 7 homolog A-like (LOC106348206)	6465
	C06	17059033 - 17060582	*Brassica napus* uncharacterized protein C23H3.12c-like (LOC106351964)	1550
	C06	17062841 - 17065619	*Brassica napus* uncharacterized (LOC106418665)	2779
**Bn-scaff_18439_1-**	C06	17104875 - 17105240	*Camelina sativa* F-box protein At1g53790-like (LOC109129984)	366

**p315543**				
	C06	17098959 - 17100106	*Brassica rapa* splicing factor U2af large subunit B-like (LOC103869103)	1148
	C06	17095805 - 17096755	*Brassica napus* RING-H2 finger protein ATL60 (LOC106417669)	951
**Bn-scaff_16903_1-p230137**	C06	20454601 - 20456043	*Brassica napus* UDP-glucose 6-dehydrogenase 4 (LOC106406052)	1443
	C06	20459481 - 20460423	*Brassica napus* S-locus-specific glycoprotein S13-like (LOC106400734)	943
	C06	20445368 - 20446705	*Brassica napus* BAG family molecular chaperone regulator 8, chloroplastic (LOC106404013)	1338
	C06	20470719 - 20472742	*Brassica napus* uncharacterized (LOC125589179)	2024
**Bn-scaff_15818_1-p292872**	C06	21078931 - 21080645	*Brassica napus* WAT1-related protein At5g40240-like (LOC106404891)	1715
	C06	21081034 - 21082624	*Brassica napus* WAT1-related protein At5g40240-like (LOC106405226)	1591
	C06	21083887 - 21084951	*Brassica napus* RING-H2 finger protein ATL46-like (LOC106402662)	1065
	C06	21085835 - 21086538	*Brassica napus* homeobox-leucine zipper protein HAT5-like (LOC106404573)	704
	C06	21089201 - 21091885	*Brassica napus* PWWP domain-containing protein 3-like (LOC125588797)	2685
**Bn-A07-p7118584**	C07	313274 - 314260	*Brassica napus* uncharacterized (LOC125583117)	987
	C07	293827 - 294433	*Brassica napus* uncharacterized (LOC106356270)	607
	C07	274607 - 275661	*Brassica napus* nudix hydrolase 25-like (LOC111214017)	1055
**Bn-scaff_17972_1-p361917**	C07	23289987 - 23291617	*Brassica napus* xyloglucan O-acetyltransferase 2-like (LOC111208226)	1631
	C07	23288704 - 23289252	*Brassica napus* uncharacterized (LOC111208228)	549
	C07	23278374 - 23279945	*Brassica napus* uncharacterized (LOC125575159)	1572
	C07	23303332 - 23306445	*Brassica napus* xyloglucan glycosyltransferase 4 (LOC111207839)	3114
	C07	23267470 - 23270149	*Brassica napus* WAT1-related protein At3g28100-like (LOC106415515)	2680
**Bn-scaff_18520_1-p117612**	C07	24204059 - 24206920	*Brassica napus* cytochrome P450 85A2 (LOC106410433)	2862
	C07	24196768 - 24196968	*Brassica napus* uncharacterized (LOC106409595)	201
	C07	24219966 - 24221596	*Brassica napus* MYB-like transcription factor EOBII (LOC125600725)	1631

	C07	24189622 - 24191193	*Brassica napus* F-box protein At2g16365 (LOC106410776)	1572
			**ADF + NDF**	
**Bn-A04-p11453319**	A04	14374351 - 14376222	*Brassica napus* uncharacterized (LOC106380565)	1872
**Bn-A04-p11463316**	A04	14369110 - 14372194	*Brassica napus* G-type lectin S-receptor-like serine/threonine-protein kinase At1g11280 (LOC106410519)	3085
	A04	14383556 - 14384125	*Brassica napus* RING-H2 finger protein ATL34 (LOC106377696)	570
	A04	14385197 - 14385793	*Brassica rapa* RING-H2 finger protein ATL60-like (LOC103864838)	597
	A04	14354343 - 14355380	*Brassica napus* probable sugar phosphate/phosphate translocator At2g25520 (LOC106420801)	1038
**Bn-A04-p13461501**	A04	16104910 - 16106124	*Brassica napus* mitoferrin (LOC106434854)	1215
	A04	16101508 - 16102086	*Brassica napus* mavicyanin (LOC106410026)	579
	A04	16108138 - 16108617	*Brassica napus* E3 ubiquitin-protein ligase RHA1B (LOC106352050)	480
	A04	16111986 - 16114214	*Brassica napus* malonyl CoA-acyl carrier protein transacylase (LOC106348411)	2229
	A04	16120073 - 16122141	*Brassica napus* laccase-3 (LOC106352049)	2069
**Bn-A04-p13705760**	A04	16347178 - 16349728	*Brassica napus* acyl-coenzyme A thioesterase 4, mitochondrial (LOC106348382)	2551
	A04	16343883 - 16346757	*Brassica napus* GTPase-activating protein GYP1-like (LOC106348383)	2875
	A04	16350820 - 16351302	*Brassica napus* glutathione S-transferase T3-like (LOC106362667)	483
	A04	16354412 - 16355338	*Brassica napus* B3 domain-containing protein At2g24670-like (LOC106426374)	927
	A04	16340178 - 16342318	*Brassica napus* uncharacterized GPI-anchored protein At1g61900-like (LOC125608070)	2141
**Bn-A04-p13726056**	A04	16371387 - 16376963	*Brassica napus* DExH-box ATP-dependent RNA helicase DExH6 (LOC106348377)	5577
	A04	16368358 - 16370259	*Brassica napus* pentatricopeptide repeat-containing protein At2g30780 (LOC106348378)	1902
	A04	16380470 - 16381213	*Brassica napus* peptidyl-prolyl cis-trans isomerase CYP19-1-like (LOC125585253)	744
	A04	16382127 - 16384632	*Brassica napus* protein JASON-like (LOC125594198)	2506
**Bn-scaff_16517_1-p354305**	A04	16371387 - 16376963	*Brassica napus* DExH-box ATP-dependent RNA helicase DExH6 (LOC106348377)	5577
	A04	16368358 - 16370259	*Brassica napus* pentatricopeptide repeat-containing protein At2g30780 (LOC106348378)	1902
	A04	16380470 - 16381213	*Brassica napus* peptidyl-prolyl cis-trans isomerase CYP19-1-like (LOC125585253)	744

	A04	16382127 - 16384632	*Brassica napus* protein JASON-like (LOC125594198)	2506
**Bn-scaff_17326_1-p536514**	C07	458840 - 461533	*Brassica napus* CBL-interacting serine/threonine-protein kinase 23-like (LOC125574997)	2694
	C07	464954 - 466652	*Brassica napus* putative tyrosine-protein phosphatase auxilin (LOC111211298)	1699
	C07	483996 - 485103	*Brassica rapa* uncharacterized (LOC103873967)	1108
	C07	507501 - 509093	*Brassica napus* phospholipase A1-Igamma1, chloroplastic-like (LOC106394391)	1593
			**Protein + Oil**	
**Bn-scaff_17441_1-p573618**	A05	26956682 - 26956975	*Brassica napus* phosphoglucan phosphatase LSF2, chloroplastic (LOC106452641)	294
	A05	26957989 - 26959276	*Brassica napus* superoxide dismutase [Mn] 1, mitochondrial-like (LOC106452637)	1288
	A05	26959802 - 26961016	*Brassica napus* reticulon-like protein B16 (LOC106452634)	1215
	A05	26948843 - 26950567	*Brassica napus* adenine/guanine permease AZG1 (LOC106452639)	1725
	A05	26969656 - 26970677	*Brassica napus* putative leucine-rich repeat-containing protein DDB_G0290503 (LOC106454248)	1022
**Bn-scaff_18356_1-p272201**	C03	44857673 - 44862288	*Brassica napus* fatty acid amide hydrolase-like (LOC125575627)	4616
	C03	44865514 - 44866284	*Brassica rapa* alkane hydroxylase MAH1 (LOC108871123)	771
	C03	44873316 - 44874773	*Brassica napus* leucine-rich repeat extensin-like protein 3 (LOC106348051)	1458
	C03	44876469 - 44881065	*Brassica napus* rDNA transcriptional regulator pol5-like (LOC125583561)	4597
			**Protein + ADF + NDF**	
**Bn-A04-p11449464**	A04	14369110 - 14372194	*Brassica napus* G-type lectin S-receptor-like serine/threonine-protein kinase At1g11280 (LOC106410519)	3085
	A04	14374351 - 14376222	*Brassica napus* uncharacterized (LOC106380565)	1872
	A04	14383556 - 14384125	*Brassica napus* RING-H2 finger protein ATL34 (LOC106377696)	570
**Bn-scaff_16755_1-p436280**	A08	485555 - 487460	*Brassica napus* uncharacterized (LOC111212106)	1906
	A08	488208 - 489776	*Brassica rapa* uncharacterized (LOC103832591)	1569
	A08	497255 - 497461	*Brassica napus* eukaryotic translation initiation factor 4E-1 (LOC106430633)	207
	A08	480584 - 480799	*Brassica napus* uncharacterized (LOC111213159)	216

**Bn-scaff_21496_1-p191960**	C05	4991203 - 5002665	*Brassica napus* uncharacterized (LOC106369956)	11463
	C05	5018386 - 5019544	*Brassica napus* zinc finger protein JAGGED-like (LOC106351233)	1159
	C05	4984961 - 4986553	*Brassica napus* berberine bridge enzyme-like 2 (LOC106400178)	1593
	C05	4973962 - 4983375	*Brassica napus* ankyrin repeat domain-containing protein 13C-like (LOC106371761)	9414
